# Microbiota in cancer: molecular mechanisms and therapeutic interventions

**DOI:** 10.1002/mco2.417

**Published:** 2023-11-05

**Authors:** Zhou Chen, Defeng Guan, Zhengfeng Wang, Xin Li, Shi Dong, Junjun Huang, Wence Zhou

**Affiliations:** ^1^ The First Clinical Medical College Lanzhou University Lanzhou Gansu China; ^2^ The First Hospital of Lanzhou University Lanzhou Gansu China; ^3^ The Second Clinical Medical College Lanzhou University Lanzhou Gansu China; ^4^ The Department of General Surgery Lanzhou University Second Hospital Lanzhou Gansu China

**Keywords:** microbiota, molecular mechanisms, oncolytic virotherapy, targeted therapy, tumor microenvironment

## Abstract

The diverse bacterial populations within the symbiotic microbiota play a pivotal role in both health and disease. Microbiota modulates critical aspects of tumor biology including cell proliferation, invasion, and metastasis. This regulation occurs through mechanisms like enhancing genomic damage, hindering gene repair, activating aberrant cell signaling pathways, influencing tumor cell metabolism, promoting revascularization, and remodeling the tumor immune microenvironment. These microbiota‐mediated effects significantly impact overall survival and the recurrence of tumors after surgery by affecting the efficacy of chemoradiotherapy. Moreover, leveraging the microbiota for the development of biovectors, probiotics, prebiotics, and synbiotics, in addition to utilizing antibiotics, dietary adjustments, defensins, oncolytic virotherapy, and fecal microbiota transplantation, offers promising alternatives for cancer treatment. Nonetheless, due to the extensive and diverse nature of the microbiota, along with tumor heterogeneity, the molecular mechanisms underlying the role of microbiota in cancer remain a subject of intense debate. In this context, we refocus on various cancers, delving into the molecular signaling pathways associated with the microbiota and its derivatives, the reshaping of the tumor microenvironmental matrix, and the impact on tolerance to tumor treatments such as chemotherapy and radiotherapy. This exploration aims to shed light on novel perspectives and potential applications in the field.

## Graphical abstract

This article presented the role of microbiota in pan‐cancer from multiple aspects, including from molecular mechanisms to therapeutic interventions, in order to provide some suggestions for future research on tumor mechanism and clinical treatment.

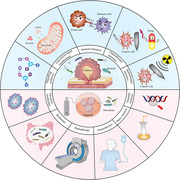

## 1 INTRODUCTION

All microorganisms in and on the surface of the human body, including bacteria, archaea, fungi, protists, and viruses, are collectively referred to as the microbiota, and 99% of them are found in the human gastrointestinal tract.^1^, ^2^ These microbiota genomes contain at least 150 times more genes than our own genomes.^3^ The microbiota is capable of coexisting with host cells at multiple body sites, and is involved in various functions such as nutrient and drug metabolism, vitamin synthesis, immunomodulation, and maintenance of gastrointestinal structures.^4^ Several epidemiologic studies have shown that specific gut microbial species are associated with an increased risk of various cancers.^5^ Recently, it has been found that a large number of microorganisms are present in tumor tissues, some of which are involved in tumorigenesis and progression.^6^ However, the molecular mechanisms remain elusive.

In fact, the intersection of microbiota and tumors goes back a long way. In the 13th century, Peregrine Laziosi suffered from osteosarcoma, which spontaneously disappeared after a severe bacterial infection.^7^ At the end of the 19th century, American surgeon William Coley observed that inducing fever could potentially cause tumors to subside.^8^ He developed a bacterial vaccine containing two killed bacteria: *Streptococcus pyogenes* and *Serratia marcescens*, known as “Coley's toxins,” which caused tumors to shrink in many of his patients.^9^ With the widespread use of antibiotics during and after surgery, the rate of postoperative infection has been effectively reduced, coupled with the routine use of antipyretics to eliminate the uncomfortable symptoms of an immune response. As a result, spontaneous remission has become less commonly reported, although when it occurs, it is often associated with acute infection.^8^ For example, a retrospective study by Ruckdeschel et al.^10^ found a significantly higher 5‐year survival rate in patients who developed pustular chest after lung cancer surgery. In 1911, a cancer‐causing virus capable of being transmissible in chicken sarcoma was discovered.^11^ In 1964, human herpesvirus 4 (HHV4) was first identified by electron microscopy in cells cultured from Burkitt's lymphoma, marking the beginning of human tumor virology.^12^ In 1970, hepatitis B virus (HBV) was discovered and was quickly linked to hepatocellular carcinoma (HCC) development.^13^, ^14^ Subsequently, the human papillomaviruses (HPV) in the etiology of cervical cancer attracted attention.^15^

**FIGURE 1:**
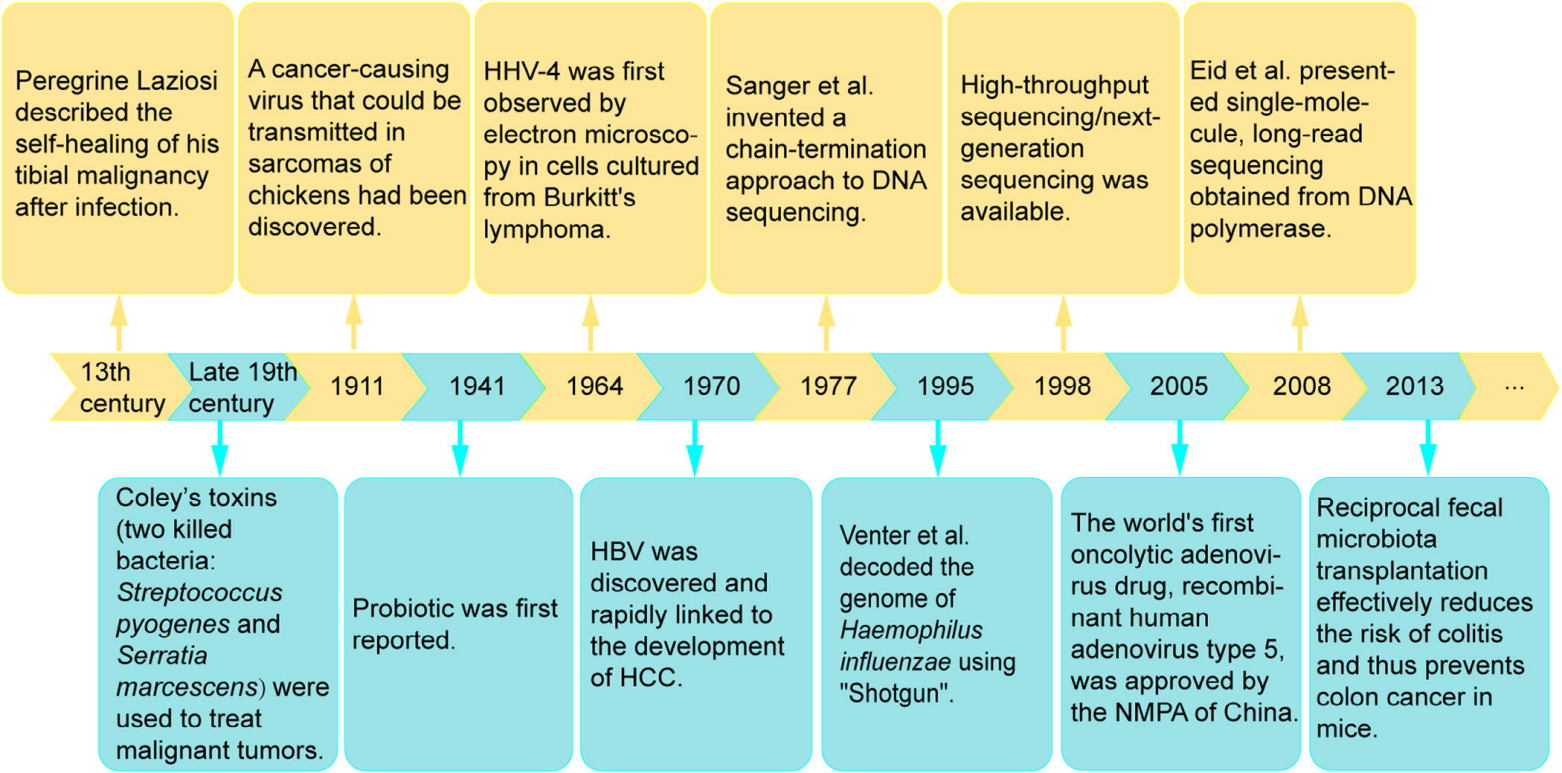
Timeline of the history and milestones of microbiota research.

Microorganisms are so diverse and numerous that it is necessary to identify strains with precision. In 1977, Sanger et al.^16^ invented a chain‐termination approach to DNA sequencing that opened the door to reading the genetic code of life. However, first‐generation sequencing can only analyze microbial diversity for bacteria that can be isolated and cultured in environmental samples, and cannot detect nonculturable microorganisms. In 1995, Venter and coworkers^17^ decoded the genome of first free‐living organism, *Haemophilus* (*H*.) *influenzae*, using his discovery of the whole‐genome sequencing method “Shotgun.” This sequencing method is flawed, the process is cumbersome and expensive, and it cannot be used to clone genes that are toxic to the host. The introduction of high‐throughput sequencing/next‐generation sequencing in 1998 overcame the inability to clone such genes, shifting from biology to chemistry to generate templates while also dramatically increasing throughput.^18^, ^19^ However, the increase in throughput comes at the cost of read length, which requires tens to hundreds of base pairs per read. This means that new short‐read techniques cannot provide the complete bacterial genome. In 2008, Eid et al.^20^ presented single‐molecule, long‐read sequencing obtained from DNA polymerase, which can produce unprecedentedly high‐quality genome assemblies. Compared with generation 1 and 2 sequencing, single‐molecule sequencing technology has the advantages of higher throughput relatively inexpensive instruments and reagents, besides its simplicity. The sequence‐based analysis provides unexpected insights into microbial diversity (from strains to superclades) and allows us to explore microbial communities.^21^, ^22^ Because of improvements in sequencing technology, some pathogenic and probiotic bacteria have been identified. Probiotic was first reported in 1941.^23^ Since then, some evidence has shown that probiotics can reduce the risk of colon cancer in humans.^24^ Reciprocal fecal microbiota transplantation (FMT) effectively reduces the risk of colitis and thus prevents colon cancer in mice.^25^ The key research milestones of intratumoral microbiota were retrospectively summarized in Figure 1. Microbiota, an emerging area of research, has been identified to be present in numerous malignancies, including breast, lung, ovarian, pancreatic, melanoma, bone, and brain tumors,^6^ and promotes efficient proliferation and evasion of cancer cell surveillance from the immune system by maintaining proliferation, evading cell growth inhibition, activating invasion and metastasis pathways, enabling replicative immunity, inducing angiogenesis, and resisting autophagy.^26^ We endeavor to provide a comprehensive and multifaceted elucidation of the relationship between the microbiota and cancer. This encompasses oncogenic causality at the genetic level, molecular mechanisms and signaling pathways, malignant behavioral shifts in tumor cells, activation of tumor stromal cells, modifications in angiogenesis, restructuring of the tumor microimmune system, tolerance to treatments like immunotherapy and chemoradiotherapy, and ultimately, the exploration of potential therapeutic prospects.

## 2 MICROBIOTA AND TUMORIGENESIS

Some microbiota in close contact with different tumors are listed in Table 1. These tumors are colonized by microbiota except for gliomas that do not readily colonize the blood–brain barrier, and most pathogenic bacteria are obligate and facultative, which are better adapted to the tumor hypoxic microenvironment. Intratumor bacteria are present in the cytoplasm of cancer and immune cells’ cytoplasms, whereas lipopolysaccharide (LPS) is present in the cytoplasm and nucleus.^6^ Currently, the microbiota promotes tumor cell transformation and tumor growth by increasing genomic damage, inhibiting gene repair, and activating aberrant cell signaling pathways.

**TABLE 1:** Studies of microbiota related to various tumors.

Cancer type	Sample size (tumor group vs. nontumor group)	Microbiome specimen	Microbial alterations (increases)	Microbial alterations (decreases)	Country	References
Glioma	27, 41	Fecal	*Fusobacterium; Akkermansia*	*Lachnospira; Agathobacter; Bifidobacterium*	China	^27^
Oral squamous cell carcinoma	50, 50	Oral tissue samples	*Aggregatibacter segnis, Campylobacter rectus, Capnocytophaga leadbetteri, Catonella morbi, Fusobacterium nucleatum, Gemella morbillorum, Peptococcus sp. Peptostreptococcus stomatis, Porphyromonas catoniae, Prevotella intermedia*	Corynebacterium matruchotii, Granulicatella adiacens, Granulicatella elegans, Streptococcus oralis	China	^28^
Lung cancer	39, 46 31, 165 25, 16	Lower airway samples	*Prevotella, Veillonella, Streptococcus* *Thermus* *Streptococcus*	– *Ralstonia* –	USA USA Spain	^29^, ^30^, ^31^
Gastric cancer	54, 81	Gastric mucosa or gastric biopsies	*Non‐Helicobacter Proteobacteria, Actinobacteria, Firmicutes*	*Bacteroidetes, Fusobacteria*	Portugal	^32^
HCC	46, 28	Tumor and normal adjacent tissues	*Stenotrophomonas, Phyllobacterium, Enterococcus_durans, Sphingomonas_leidy*	Acute myeloid leukemia; Nasopharyngeal carcinoma	China	^33^
Intrahepatic cholangiocarcinoma	45, 52	Tumor and paracancerous tissues	*Ralstonia pickettii, Acinetobacter johnsonii*	–	China	^34^
Extrahepatic cholangiocarcinoma	100, 100	Biliary duct epithelial cells	*Methylophilaceae, Fusobacterium, Prevotella, Actinomyces, Novosphingobium, Helicobacter pylori*	*Nesterenkonia*	Mexico	^35^
Gallbladder cancer	10, 5	Bile	*Klebsiella*	–	Korea	^36^
Pancreatic ductal adenocarcinoma	13, 5 12, 5	Pancreatic tissue specimens	*Malassezia; Proteobacteria; Bacteroidetes; Firmicutes*	–	USA	^37^, ^38^
Colorectal cancer	184, 204 89, 161	Fecal	*Ascomycota, Malasseziomycetes; Bacteroides, Fusobacterium, Dorea, Porphyromonas*	*Saccharomycetes, Pneumocystidomycetes; Pseudomonas, Prevotella, Acinetobacter, Catenibacterium*	China Korea	^39^, ^40^
Breast cancer	221, 87	Breast specimens	*Pseudomonadaceae, Enterobacteriaceae, Proteus, Porphyromonas, Azomonas*	*Stenotrophomonas, Caulobacter*	USA	^41^
Renal cell carcinoma	24, 24	Cancer samples and adjacent normal tissue samples	*Nitriliruptor, Deinococcus, Actinomyces, Gordonia, Pseudoclavibacter, Microlunatus Amycolatopsis, Weissella, Brevundimonas, Phyllobacterium*	–	China	^42^
Endometrial cancer	17, 10	Uterus and lower genital tract samples	*Anaerostipes, Ruminococcus, Atopobium, Bacteroides, Porphyromonas, Dialister*	–	USA	^43^
Cervical cancer	60, 60	Cervicovaginal fluid samples	*Streptococcus urinalis, Escherichia coli, Bacillus safensis, Bacillus malikii, Corynebacterium jeikeium, Corynebacterium striatum, Lactobacillus rhamnosus*	*Staphylococcus pasteuri, Staphylococcus auricularis, Staphylococcus capitis subsp. capitis, Facklamia hominis, Paenibacillus urinalis, Pseudocitrobacter faecalis, Brevibacterium masiliense, Klebsiella oxytoca*	Mexico	^44^
Ovarian cancer	176, 184	Cervical smear samples	*Gardnerella, Atopobium*	–	UK	^45^
Bone cancer	39, –	Bone	*Sphingomonas yanoikuyae, Actinomyces massiliensis, Pseudomonas argentinensis, Enterobacter asburiae*	–	Israel	^6^

### 2.1 Microbiota and increased genomic damage

#### 2.1.1 Carcinogens produced by pathogenic bacteria

The microbiota can produce proteins, molecules, and secondary metabolites that interact directly with host cell DNA, thereby mutating it.^46^ Pathogenic *Escherichia (E.) coli* secretes genotoxic colibactin, and once internalized by colon cancer cells, they can induce DNA double‐strand breaks (DSBs), promoting tumor cell transformation and mutations in multiple cancer genomes.^26^, ^47^, ^48^ In human cell cultures, colibactin is synthesized by enzymes encoded by genotoxic island polyketide synthase (*pks*) and is thought to cause interstrand crosslinks by alkylating DNA on adenine residues.^49^, ^50^, ^51^, ^52^ Deletion of *pks* from *E. coli* NC101 reduced tumor multiplicity and invasion in azoxymethane (AOM)‐treated interleukin‐10‐deficient mice.^52^, ^53^ Some strains of *E. coli* yield toxins called cyclomodulins that alter host cells'eukaryotic cell cycle.^54^ In addition to the induced DNA damage response, *E. coli* indirectly affects colorectal carcinogenesis through its effect on Wnt signaling.^55^, ^56^ *Campylobacter* (*C*.) *jejuni* produces a genotoxin with DNA enzyme activity, the cytolethal distending toxin (CDT), causing DNA DSBs, which induces colorectal cancer (CRC) in vivo.^57^ CDT restricted to epsilon and gamma categories in phylum *Proteobacteria*.^58^ CDT is a heteropolymeric protein consisting of three subunits, CdtA, CdtB, and CdtC, that primarily causes G2/M phase block in epithelial, fibroblast, and lymphocyte cells. CdtB has been shown to be a toxic subunit, promoting DSBs in vitro and in vivo, and CdtA and CdtC are essential for CdtB delivery.^59^, ^60^, ^61^, ^62^, ^63^ At low to moderate doses (50 pg mL^−1^), CdtB causes DNA single‐strand breaks (SSBs), which are resolved by SSB repair.^64^ At a high dose (over 1 μg mL^−1^), CDT can directly induce DNA DSBs (during the S‐phase) when numerous SSBs are facing each other on different DNA strands.^65^ *F. nucleatum* is an oral bacterium that produces hydrogen sulfide (H_2_S) from l‐cysteine via the enzymatic activity of l‐cysteine desulfurase, thereby increasing DNA damage.^66^, ^67^ Furthermore, colonic bacteria, including *Bilophila (B.) wadsworthia* and *Desulfovibrio* (*D*.) *desulfuricans*, can produce H_2_S.^68^ There is evidence that the production of H_2_S leads to DNA damage in part due to the production of reactive oxygen species (ROS).^69^ Recent studies found that indolimines‐produced by the CRC‐associated species *Morganella (M.) morganii* cause DNA damage, which exacerbates colon tumorigenesis in mice.^70^ Enterotoxigenic *Bacteroides* (*B*.) *fragilis* (ETBF) encodes *B. fragilis* toxin (BFT), which increases inflammation and intestinal permeability by targeting enterocyte tight junctions and cleaving E‐calcine mucin and triggers Wnt/β‐catenin and nuclear factor‐kappaB (NF‐κB) nuclear signaling in intestinal epithelial cells, contributing to oncogenic transformation in the colon.^71^, ^72^ ETBF infection may promote CRC tumorigenesis by upregulating epigenetic and transcriptional regulators in the Toll‐like receptor 4 (TLR4)‐dependent pathway, impacting stemness regulation.^73^ The type III secretion system (T3SS) transfers effector proteins, including inactive typhoid toxin, and the AvrA targets cells involved in tumorigenesis through genotoxin‐mediated mutagenesis and allow intracellular bacteria to survive and favor ecological dysregulation.^74^ AvrA stimulates JAK/STAT signaling and Wnt/β‐linked protein activation, cell proliferation, and differentiation, as well as enhancing acetyltransferase activity that targets p53, which together drive apoptosis inhibition besides cell cycle arrest, ultimately carcinogenesis.^74^ Mitogen‐activated protein kinase (MAPK) and AKT signaling activation is essential for maintaining cellular transformation in gallbladder cancer in mouse models during infection with *Salmonella (S.) typhimurium* in cells.^75^

#### 2.1.2 Carcinogens produced by dietary metabolites of pathogenic bacteria

High protein consumption raises colon protein levels, allowing *Firmicutes* and *Bacteroides* to ferment amino acids into N‐nitroso compounds, causing host mutations via DNA alkylation.^76^ Colonic bacteria metabolize carcinogenic heterocyclic amines to produce DNA‐damaging agents (ethanol and heterocyclic amines) or direct carcinogens (fecpentaenes). N‐nitroso compounds that ferment amino acids.^77^ *Clostridium* (*C*.) *scindens* and other bacteria convert primary bile acid into secondary deoxycholic acid (DCA). DCA acts as a tumor promoter by disrupting cell membranes to release arachidonic acid, which is converted by cyclooxygenase‐2 (COX‐2) and lipoxygenase to prostaglandins and ROS, triggering inflammation and DNA damage. Taurine also acts as a tumor promoter through the production of genotoxic H_2_S and stimulates certain bacteria growth like *B. wadsworthia*.^78^ Additionally, the DNA‐damaging bacterial metabolite DCA was discovered to promote the development of HCC in a mouse model by inducing a senescence‐associated secretory phenotype (SASP) in hepatic stellate cell (HSC), which secretes various inflammatory and tumor‐promoting chemicals.^79^ Using a mouse model of diet‐induced obesity, Yoo et al.^80^ found that high‐fat diet (HFD) escalates *E. coli* choline metabolism by changing intestinal epithelial physiology. The HFD impaired mitochondrial bioenergetics in the colonic epithelium to surge intraluminal bioavailability of oxygen and nitrate, eventually exacerbating respiration‐dependent cholinergic metabolism in *E. coli*. In turn, *E. coli* choline catabolism increases levels of circulating trimethylamine N‐oxide (TMAO), toxic metabolite generated by intestinal microbes. Additionally, HFD stimulates the synthesis of hepatic bile acids, which, when converted to secondary bile acids in the colon, may promote tumorigenesis, which is associated with changes in the structure of the intestinal microbiota.^81^, ^82^

Furthermore, pathogenic bacteria can produce indirect carcinogens through chronic infection and inflammation, leading to carcinogenic mutations in host cells.^83^ For example, neutrophils, monocytes, and macrophages present in the inflammatory environment produce several oxidants and nitroso substances which are recognized to be genotoxic and mutagenic.^84^ This may be related to the upregulation of myeloperoxidase levels and inducible nitric oxide synthase (iNOS) by these infiltrating immune cells.^85^, ^86^ Nucleotide‐binding oligomerization domain 2 (NOD2) is a universal intracellular pattern recognition receptor (PRR) that recognizes the muramyl dipeptide present in Gram‐negative bacteria and Gram‐positive bacteria. When NOD2 is active, it translocates to the nucleus, displays nuclear translocation, and directly binds to lamin A/C, a protein component of nuclear laminae, to promote its protein degradation, which in turn reduces lamin A/C and increases DNA damage repair failure.^87^

#### 2.1.3 RNA modification

Intestinal bacteria affect the availability of methyl donor material and influence N6‐methyladenosine (m^6^A) RNA methylation. As one of the epigenetic/epigenetic transcriptome modifications, m^6^A RNA methylation is closely associated with cancer development and progression.^88^ In order to increase susceptibility, microbial pathogens can modify host m^6^A methylation, which in turn disrupts lung immune homeostasis and affects the genesis, progression, and clinical outcome of non‐small cell lung cancer (NSCLC).^89^ Downregulating the m^6^A methyltransferase in CRC cells and patient‐derived xenograft tissues are one way in which *F. nucleatum* promotes CRC invasiveness. Mechanistically, *F. nucleatum* activates yes1‐associated transcriptional regulator signaling, which inhibits forkhead box D3, a transcription factor for methyltransferase‐like 3 (METTL3), and consequently reduces METTL3 transcription. Downregulation of METTL3 promotes the expression of its target kinesin family member 26B (KIF26B) by decreasing its m^6^A level and reducing YTH domain family protein 2‐dependent mRNA degradation, which contributes to *F. nucleatum*‐induced CRC metastasis. In CRC tissues, METTL3 expression was inversely associated with *F. nucleatum* and KIF26B levels. High KIF26B expression is considerably associated with shorter survival time of CRC patients.^90^ LPS stimulation promotes G‐protein alpha‐subunit (GNAS) expression in HCC cells by increasing m^6^A methylation of GNAS mRNA. High GNAS expression level promotes LPS‐induced HCC cell growth and invasion by interacting with signal transducer and activator of transcription 3 (STAT3). GNAS knockdown suppresses LPS‐induced IL‐6 expression in HCC cells by inhibiting STAT3 activation. In addition, GNAS promotes LPS‐induced STAT3 activation in HCC cells by inhibiting the interaction of long noncoding RNA TPTEP1 with STAT3. GNAS expression triggers HCC progression among mice and is associated with low survival rates.^91^

#### 2.1.4 Virus

Viruses are clonally incorporated into the genome of tumor cells to alter gene expression. Viruses can express viral oncogenes that directly promote the transformation of cancer cells.^83^ In higher than 100 tumor samples, including colon, breast, genitourinary, and oral tumors. Viral positivity was detected in 54 (27%) of the 197 samples tested at the overlapping cluster level and in 106 (54%) of the samples tested using read mapping. All samples tested positive for the virus in numerous types of skin‐related and mucosal cancers. The viruses detected mainly belonged to the families *Papillomaviridae*, *Polyomaviridae*, *Herpesviridae*, *Parvoviridae*, and *Anelloviridae*.^92^ To date, seven viruses that infect humans including HBV, hepatitis C virus (HCV), human T‐lymphotropic virus (HTLV), HPV, Epstein–Barr virus (EBV), Kaposi's sarcoma herpesvirus (KSHV), and Merkel cell polyomavirus (MCV) have been universally recognized as causes of cancer.^93^ In almost all HBV‐associated cancers, HBV is clonally integrated into the genome of tumor cells.^83^ Transgenic models showed that various proteins from these viruses, including the HBV‐encoded X protein, the HCV‐encoded nonstructural protein, and the HTLV‐I encoded Tax, can initiate oncogenic transformation.^94^, ^95^ HPV is primarily detected in skin and mucosa‐associated cancers.^96^ The oncoproteins encoded by the HPV E6 and E7 oncogenes are important factors leading to cervical epithelial carcinogenesis, and the combination of these two proteins with the intracellular oncoproteins p53 and pRb can markedly alter the cellular growth cycle and DNA repair, which consequently leads to genomic instability.^97^ EBV, also known as HHV4, was the first human tumor virus to be discovered.^12^ EBV has been found to be associated with a variety of tumors of lymphoid and epithelial origin.^98^ EBV promotes cancer development by inducing host genome instability, altering the epigenetic profile of the host genome, assisting cancer cells in evading the immune response, promoting cancer cell survival as well as inducing stem cell‐like properties.^99^ KSHV, also known as HHV8, is the causative agent of Kaposi sarcoma and promotes KSHV‐associated B‐cell lymphocyte proliferation through the expression of several viral proteins, such as the latency‐associated nuclear antigen, viral interferon (IFN) regulatory factor 3 (IRF3), viral IL‐6, viral cyclin, and viral FLICE‐inhibitory protein.^100^ MCV is the causative agent of most Merkel cell carcinoma and maintains proliferative signaling by expressing two viral proteins large T antigen and small T antigen through inhibition of RB and p53, respectively.^101^ Besides that, whether the virus plays a role in inducing angiogenesis and avoiding the immune response needs to be further investigated.

### 2.2 Microbiota and DNA repair

Throughout the cell cycle, human DNA undergoes repetitive DNA damage events. A complex network of cellular systems exists to maintain the integrity of DNA. This biological system detects DNA damage and promotes DNA cell cycle repair checkpoints or apoptosis, or both. The process of DNA mismatch repair (MMR) is a well‐studied and widely used biological route that helps keep genes intact and the genome stable. DNA replication and recombination both generate base mismatches and insertion/deletion mismatches, which are specifically targeted by MMR.^102^ For instance, enzyme activation‐induced cytidine deaminase (CDD) converts cytosine to uracil, creating a G:U mismatch detected and treated by MMR.^103^, ^104^ MutL homolog 1 (MLH1) and mutS homolog 2 (MSH2) play central roles in the MMR. Both form heterodimeric complexes with all other MutL and MutS homologs, respectively, and deletion of MSH2 or MLH1 results in complete inactivation of the MMR in humans and other species.^105^

*E. coli* depletes the MMR proteins MSH2 and MLH1 in cultured colon cells in a T3SS‐dependent manner, inducing host mutagenesis.^106^, ^107^ Similar results were seen in *Helicobacter* (*H*.) *pylori*‐infected GEpiC.^108^ This may be due to specific CagA EPIYA motifs and vacA genotypes.^109^ *H. pylori* suppress MMR protein (MLH1, MSH2, MSH3, and MSH6) expression through miR‐155‐5p and miR‐3163 upregulation.^110^ *F. nucleatum* increases miR‐205‐5p expression through TLR4 and myeloid differentiation primary‐response protein 88 (MyD88)‐dependent innate immune signaling pathways and suppresses MLH1, MSH2, and MSH6 expression, leading to DNA MMR damage and cell proliferation in HNSC.^111^ Among the 30 cancer types, validation revealed 21 different mutational signatures, with C>T at NpCpG mutations predominating in Signature 6. This pattern of indels (mostly of 1 bp) is commonly referred to as “microsatellite instability (MSI).”^112^ MSI is a hypermutated phenotype resulting from deficient DNA MMR activity, prevalent in around 15% of CRC. Out of these cases, Lynch syndrome accounts for only 3%, while the remaining 12% are linked to sporadic acquired hypermethylation of MLH1 gene promoter observed in tumors with CpG island methylator phenotype.^113^, ^114^

**FIGURE 2:**
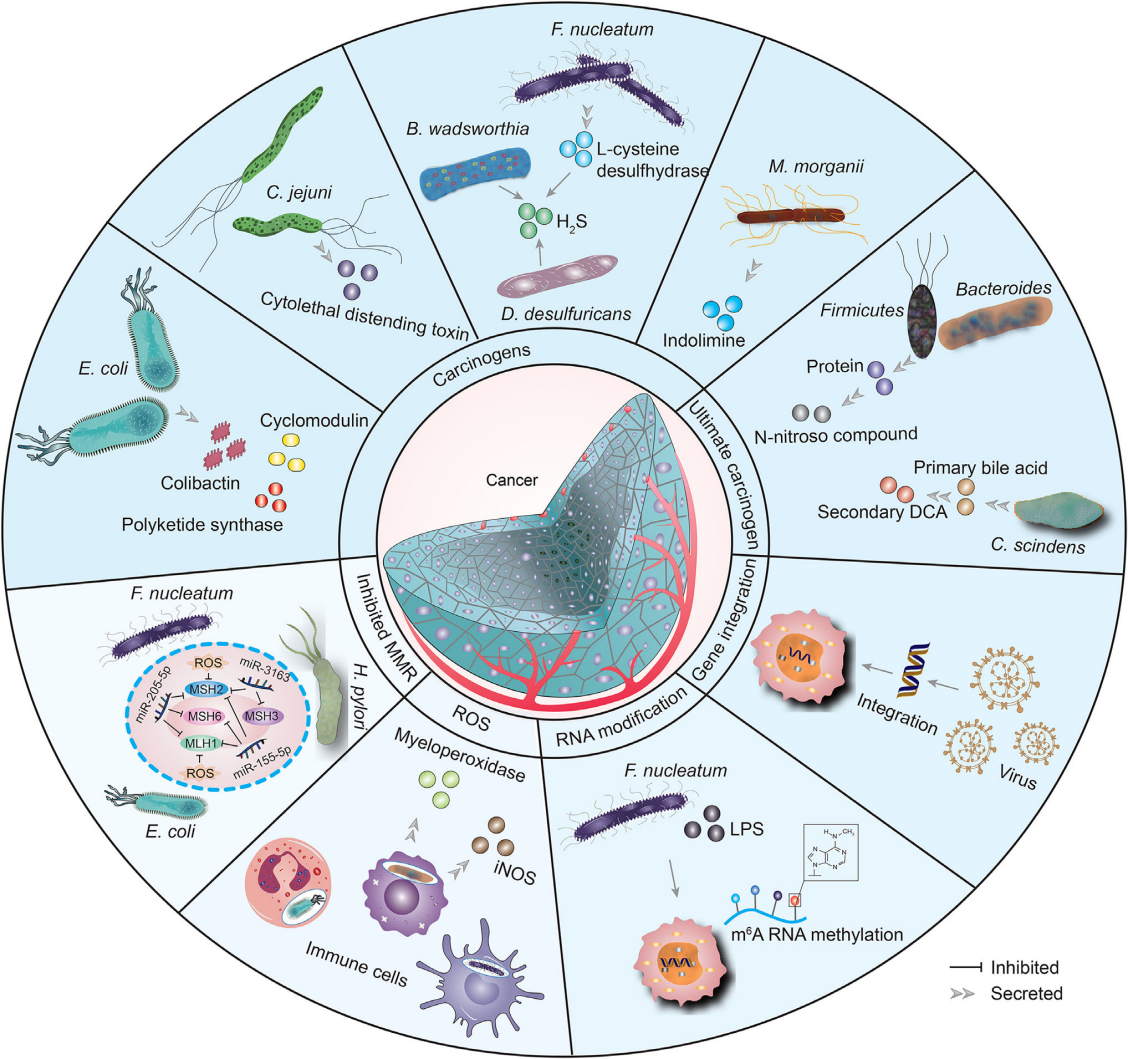
Microbiota in TME increases tumorigenicity. Microbiota damage host cell genes by secreting carcinogens (e.g., cyclomodulin, colibactin, polyketide synthase, cytolethal distending toxin, H_2_S, and indolimine), as well as ultimate carcinogen (e.g., N‐nitroso compound and secondary DCA). The virus becomes integrated into the genome of tumor cells, leading to alterations in gene expression. Microbial pathogens enhance the malignant behavior of cancer cells by modifying host m^6^A modifications. The microbiota recruits immune cells to secrete myeloperoxidase and iNOS, leading to increased ROS production and exacerbating genetic damage in the host. Additionally, a concurrent presence of microbiota inhibits gene repair pathways, such as those associated with MMR proteins (including MSH2, MSH6, and MLH1). DCA deoxycholic acid, iNOS inducible nitric oxide synthase, ROS reactive oxygen species, MSH2 mutS homolog 2, MSH6 mutS homolog 6, MLH1 mutL homolog 1.

In addition to changes in gut microbiota richness and diversity, elevated levels of *Proteobacteria* were associated with decreased HR of thyroid carcinoma by typing analysis, a mechanism that needs to be further investigated.^115^ HFD leads to disruption of the gut microbiota, causing innate immune dysregulation, producing an inflammatory environment, decreasing DSB repair, and being reversed with antibiotic treatment.^116^ The microbiota in a hypoxic microenvironment increases tumorigenesis as demonstrated in Figure 2.

### 2.3 Microbiota and signaling pathways in TME

Long‐term settlement of microbiota in tumor tissue, on the one hand, causes an inflammatory response, which can result in damage to the vascular endothelium, dysfunction of vascular endothelial cells, impaired blood transport, leading to hypoxia. On the other hand, some pathogenic bacteria can rob oxygen from the TME, or microbial metabolites, such as short‐chain fatty acids (SCFAs), increase cellular O_2_ consumption through β‐oxidation of butyrate and oxidative phosphorylation (OXPHOS), thereby stabilizing hypoxia‐inducible factor (HIF).^117^, ^118^, ^119^ Moreover, microbiota‐recruited innate and adaptive immune cell infiltrates, most notably infiltrating neutrophils and eosinophils, consume local oxygen via the nicotinamide adenine dinucleotide phosphate (NADPH) oxidase during respiratory burst.^120^, ^121^ A large amount of ROS produced by these inflammatory responses stimulates HIF‐1α expression and becomes stable under hypoxic conditions.^122^ TLRs are a type of PRR that has a crucial function in the immunological host defense system by identifying pathogen‐associated molecular patterns.^123^ After LPS stimulation, splicing protein like MyD88 is recruited to the TLR cytoplasmic structural domain.^124^, ^125^ LPS‐induced ROS production and TLR4 or MyD88 are required for HIF‐1 activation.^125^ LPS stimulates macrophages to activate HIF‐1α in TLR4‐dependent rather than hypoxia manner, increases HIF‐1α mRNA level, decreases prolyl hydroxylase (PHD) mRNA production, and promotes glucose transporter 1 (GLUT1) and vascular endothelial growth factor (VEGF) expression.^126^, ^127^ LPS increases HIF‐1α mRNA expression by stimulating the monocytes NF‐κB, while hypoxia stabilizes HIF‐1α protein after translation.^128^

**FIGURE 3:**
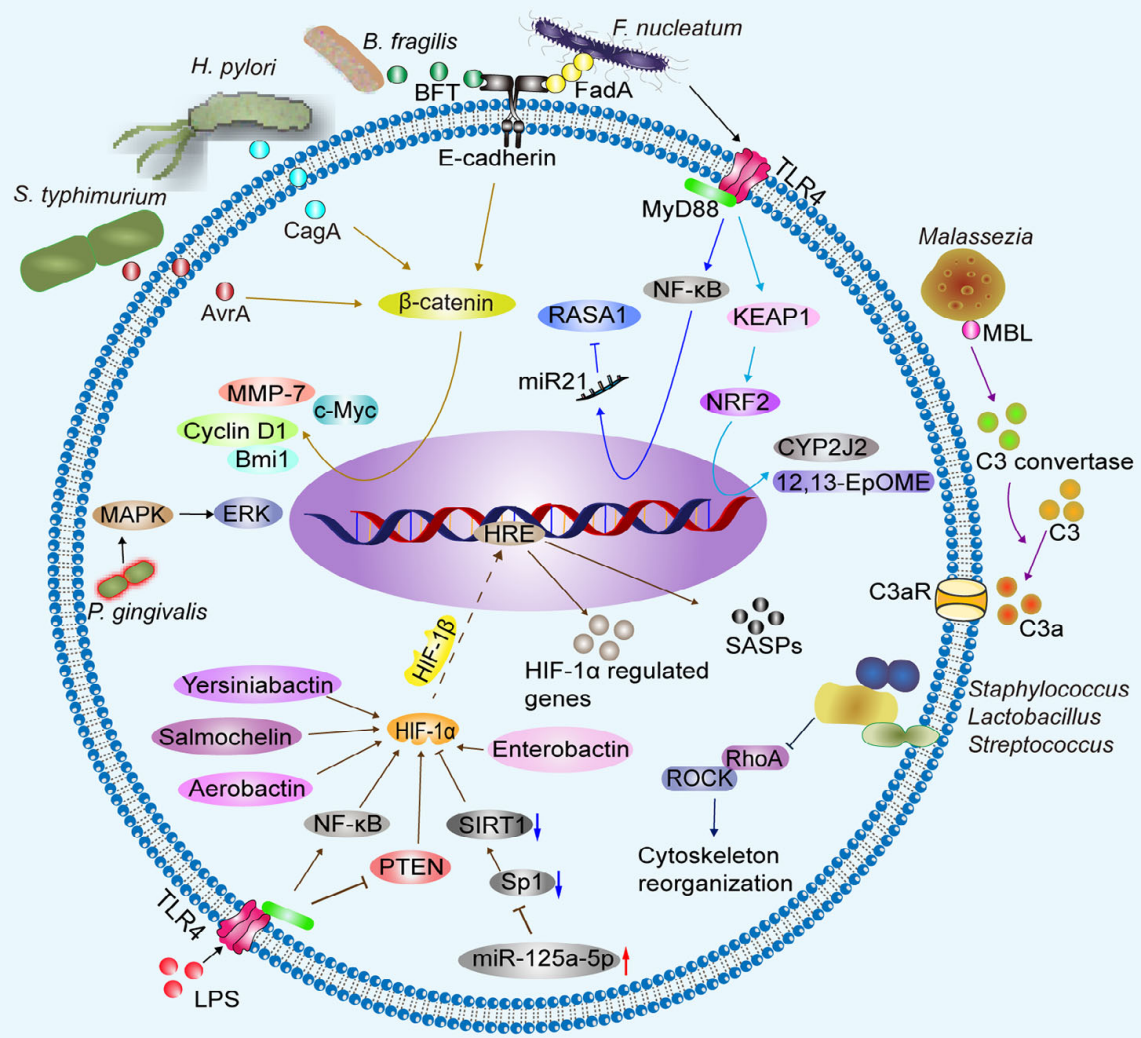
Tumor cell signaling pathways altered by microbiota. AvrA, secreted by *S. typhimurium*, and CagA, produced by *H. pylori*, can activate β‐catenin signaling. BFT, encoded by *B. fragilis*, and FadA on the surface of *F. nucleatum*, can bind to E‐cadherin and activate β‐catenin signaling, promoting the expression of downstream genes such as Bmi1, MMP‐7, cyclin D1, and c‐Myc. *F. nucleatum* activates TLR4 signaling through MyD88, which activates NF‐κB, increases miR21, and ultimately decreases RASA1. *F. nucleatum* also increases CYP2J2 and 12,13‐EpOME through activation of the TLR4/KEAP1/NRF2 pathway. MBL binds to glycans on the *Malassezia* wall, triggering C3 convertase, which cleaves C3 to C3a. *Staphylococcus*, *Lactobacillus*, and *Streptococcus* penetrate the cell membrane and localize in the cytoplasm, inhibiting RhoA and ROCK activation, and remodeling the cytoskeleton to resist mechanical stress. *P. gingivalis* can activate the MAPK/ERK signaling pathway. LPS stimulation of TLR4/MyD88 promotes HIF‐1α by activating NF‐κB or inhibiting PTEN. Microbiota exacerbate ROS and microbiota secretions such as siderophores (yersiniabactin, salmochelin, aerobactin) that stabilize HIF‐1α, which binds to HIF‐1β and enters the nucleus to bind HRE, stimulating the expression of HIF‐1α‐regulated genes. Elevated miR‐125a‐5p suppresses Sp1/SIRT1 expression and increases HIF‐1α acetylation levels, consequently raising SASP levels. MMP‐7, metalloproteinase‐7; TLR4, Toll‐like receptor 4; MyD88, myeloid differentiation primary‐response protein 88; NF‐κB, nuclear factor‐kappaB; CYP2J2, cytochrome P450 2J2; 12,13‐EpOME, 12,13‐epoxyoctadecenoic acid; KEAP1, Kelch‐like ECH‐associated protein 1; NRF2, nuclear factor‐erythroid 2‐related factor 2; MBL, mannose‐binding lectin; PTEN, phosphatase and tensin homolog deleted on chromosome ten; HIF, hypoxia‐inducible factor; HRE, hypoxia response element; SIRT1, sirtuin 1; Sp1, specificity protein 1.

LPS activates the TLR4‐NF‐κB pathway to upregulate HIF‐1α, which in turn promotes pancreatic cancer (PC) progression.^129^ Furthermore, LPS promotes PC cell migration by decreasing the levels of two tumor suppressors, phosphatase and tensin homolog deleted on chromosome ten (PTEN) and MAP2K4, through the TLR4‐miR‐181a signaling pathway.^130^ PTEN deletion promotes HIF‐1‐mediated gene expression,^131^ Siderophores (yersiniabactin, salmochelin, aerobactin) are secreted by *Yersinia enterocolitica*, *S. enterica subsp enterica*, and *Enterobacter aerogenes*, induce dose‐dependent HIF‐1 stabilization in epithelial and endothelial cells, and independently with hypoxia.^132^ Pathogenic Gram‐negative bacteria secretes enterobactin that stabilizes HIF‐1α, which binds to HIF‐1β and enters the nucleus to bind hypoxia response element, stimulating the expression of HIF‐1α‐regulated genes.^133^, ^134^ CagA produced by *H. pylori* is injected into host cells via the type IV bacterial secretion system (T4SS), which activates β‐catenin, leading to the upregulation of transcriptional genes associated with gastric cancer.^135^ Insertion of AvrA secreted by *S. typhimurium* into host cells upregulates β‐catenin signaling, characterized by increased Bmi1, matrix metalloproteinase‐7 (MMP‐7), and cyclin D1.^136^ BFT induces E‐calmodulin cleavage, β‐catenin nuclear localization, upregulation of c‐Myc transcription and translation, and cancer cell proliferation.^71^, ^137^ FadA on the surface of *F. nucleatum* binds to E‐cadherin and activates β‐catenin signaling.^138^ *F. nucleatum* activates TLR4 signaling to MyD88, which activates NF‐κB and increases the expression of miR21, a miRNA that reduces the level of the RAS GTPase RASA1.^139^ *F. nucleatum* increases cytochrome P450 2J2 and 12,13‐epoxyoctadecenoic acid through activation of the TLR4/Kelch‐like ECH‐associated protein 1/NF‐erythroid 2‐related factor 2 axis to promote epithelial–mesenchymal transition (EMT) and metastasis in CRC.^140^ *Porphyromonas* (*P*.) *gingivalis* can invade CRC cells and promote their proliferation by significantly activating the MAPK/ERK signaling pathway.^141^ Mannose‐binding lectin binds to glycans of the *Malassezia* wall and triggers C3 convertase, which can cleave C3 to C3a, and then C3a binds to C3aR on the surface of the tumor cells, promoting proliferation of PC cells in vitro and tumor growth in vivo.^37^ *Staphylococcus*, *Lactobacillus*, and *Streptococcus* penetrate the cell membrane and localize in the cytoplasm, inhibit RhoA and ROCK activation, remodel the cytoskeleton to resist mechanical stress, and promote lung metastasis of murine breast cancer cells.^142^ Tumor cell signaling pathways altered by microbiota are showed in Figure 3.

Microbiota can alter the malignant phenotype of tumors by inducing inflammation, modulating the antitumor immune system, altering TME, and influencing cellular metabolism, and is strongly linked to tumor patients' prognosis.^143^, ^144^ TLR activated by microbiota can cause derangements of multiple tumor suppressor proteins (e.g., p16, p21, p27, p53, pRb, PTEN, and MAP2K4), induce STAT3 activation, and enhance EMT and oncogene‐induced senescence.^145^ Microbiota, on the other hand, can generate ROS by inducing a prolonged inflammatory response via changes of essential substance as lipids, nucleic acids, and preproteins that result in protein misfolding, membrane disintegration, and DNA fragmentation and accumulation over time,^143^ which may dominate cellular senescence.^146^ Senescent cells, in contrast to quiescent and apoptotic cells, are nonetheless highly viable and perform metabolic functions efficiently.^147^ SASPs are a group of paracrine signaling molecules secreted by senescent cells that include cytokines, chemokines, growth factors, and proteases.^148^ In an autocrine or paracrine fashion, senescent cells promote the emergence of age‐related disorders, particularly cancers, while also aiding in tissue formation, suppressing tumors, tissue healing, and cell proliferation.^144^ Evidence suggests that xenograft tumor growth can be accelerated by a dysregulated SASP that sustains inflammatory conditions in TME that promote cancer proliferation, migration, invasion, and EMT.^144^ For instance, exposure to the harmful DCA, produced by intestinal bacteria, stimulates cellular senescence and secretes SASP, which increases tumor‐promoting inflammation and promotes the development of HCC.^79^ There are now experiments to identify the molecular mechanisms of hypoxia‐associated senescence onset. For example, elevated miR‐125a‐5p is implicated in senescence and SASP secretion across lung epithelial cells via specificity protein 1 (Sp1)/sirtuin 1 (SIRT1)/ IF‐1α.^149^ Inhibition of HIF‐1α may inhibit SASP secretion.^150^

## 3 MICROBIOTA AND TUMOR CELL METABOLISM

A common feature of tumor cell metabolism is the ability to obtain essential nutrients from nutrient‐poor environments and utilize them to maintain viability. Alterations in intracellular and extracellular metabolites accompanied by tumor‐associated metabolic reprogramming have profound effects on gene expression, cellular differentiation, and the TME. The microbiota and tumor cell metabolism (especially glycolysis and lipid metabolism) have also been widely concerned.

### 3.1 Glycolysis

Even under aerobic settings, tumor cells glycolyze glucose into lactate, called the Warburg effect, to meet their energy requirements.^151^ HIF‐1α is the primary transcription factor responsible for promoting Warburg‐like metabolism.^151^ Some bacteria also affect the glycolysis of tumor cells. Aspergillus fumigatusinduces aerobic glycolysis in colon cancer cells.^152^ Increased ENO1‐IT serve as a guide module for KAT7 histone acetyltransferase, specifying histone modification patterns of its target genes (as ENO1) and thereby varying CRC glycolysis and tumorigenesis. This is accomplished by enhancing Sp1's binding efficiency to lncRNA ENO1‐IT1 promoter region.^153^ However, some bacterial metabolic wastes such as microbiota‐derived *Staphylococcal* superantigen‐like protein 6 (SSL6) downregulate PI3K/AKT‐mediated glycolysis by hindering CD47, which contributed to enhanced susceptibility of HCC to sorafenib.^154^ Microbiota can regulate immune cell glycolysis through TLR signaling. When TLR was activated, the glycolysis of macrophages increased significantly, and mitochondrial activity was inhibited. HIF‐1α stabilization with ROS after LPS stimulation significantly upregulated GLUT1, hexokinase 3, 6‐phosphofructo‐2‐kinase/fructose‐2,6‐biphosphatase 3, phosphoglucomutase‐2, and enolase 2 (ENO2) confirming the increase in glycolysis.^155^ This shift in metabolic activity, called glycolytic reprogramming, leads to altered mitochondrial function, increased ROS production, and increased secretion of proinflammatory cytokines.^156^ LPS‐stimulated TLR signaling in immune cells (e.g., monocytes, dendritic cells [DCs], regulatory T [Treg] cells) increased PI3K–AKT–mTORC1 signaling, glycolysis, and GLUT1 expression.^157^, ^158^, ^159^ TLR also induces a metabolic shift from OXPHOS to aerobic glycolysis in DC via PI3K/Akt pathway, mTOR–iNOS–nitric oxide (NO), because NO can inhibit mitochondrial function.^160^, ^161^, ^162^ Other bacteria, such as *B. uniformis*, have a strong glycolytic capacity and produce butyrate.^163^ Butyrate accumulates in tumor cells because of the Warburg effect, where it functions as an histone deacetylase inhibitor to promote histone acetylation, which in turn suppresses cell proliferation and induces apoptosis, so helping to prevent CRC.^164^

### 3.2 Lipid metabolism

The study findings revealed that several bacterial taxa in the oral and intestinal tracts, such as *Fusobacterium*, *Erysipelotrichaceae*, and *Lachnospiraceae* families in the gut, were strongly connected to low‐density lipoprotein and total cholesterol.^165^ Compared with germ‐free mice, conventionally housed mice had significantly higher levels of pyruvate, citrate, fumarate, and malate and lower cholesterol and fatty acid (FA) levels.^166^ Microbiota provide raw materials for lipid metabolism synthesis through their own metabolites or stimulate lipid synthesis in host cells. For example, SCFAs serve as substrates for various metabolic processes, including cholesterol synthesis, lipogenesis, and gluconeogenesis.^167^ Microbiome‐derived metabolite δ‐valerobetaine activates genes encoding mitochondrial energy production and FA oxidation via the transcription factor PPAR‐α, thereby reducing mitochondrial FA oxidation and increasing lipid accumulation.^168^ Microbes induce monounsaturated FA production via stearoyl‐CoA desaturase 1 and poly‐unsaturated FA elongation with FA elongase 5, resulting in significant glycerophospholipid acyl‐chain profile alterations, as acetate from intestinal microbiota breakdown of dietary fiber is a precursor for hepatic production of C16 and C18 FAs and their related glycerophospholipid species, which are likewise transferred into circulation.^169^ Small bowel *C. bifermentans* selectively induces participation in diacylglycerol O‐acyltransferase 2, which increases duodenal and jejunal oleic acid uptake.^170^ Activation of TLR‐4 signaling may promote increased lipid synthesis.^171^ However, controversy exists.^172^ Bile acids help absorb dietary lipids and fat‐soluble vitamins. Synthesized from cholesterol in the liver, they are stored within the gallbladder before being released into the intestine postingestion. Primary bile acids combine with taurine or glycine and undergo further metabolism by *C*. (clusters XIVa and XI) as well as *Eubacterium* to produce secondary bile acids.^173^, ^174^, ^175^ About 95% of bile acids that are secreted into the intestine are taken back up, mostly as bound bile acids located in the distal ileum by a protein known as ASBT or IBAT. These reabsorbed bile acids go through the portal vein and return to the liver where they are once again released. This cycle, known as hepatic‐intestinal cycle, takes place approximately six times daily in humans.^176^ Bile acids have the capacity to serve as signaling agents by binding with farnesoid X receptor (FXR), that is a nuclear receptor predominantly present in the ileum and liver.^177^ FXR has a role in the control of lipid metabolism, namely the transportation, production, and usage of triglycerides.^178^ Chenodeoxycholic acid (CDCA) is the strongest FXR ligand, followed by cholic acid, DCA, and lithocholic acid,^176^ and in an FXR‐dependent manner, gut microbiota stimulates excess weight and hepatic steatosis,^179^ whereas in hepatic FXR absence, elevated LXR expression and induction of LXR's lipogenic target genes, Scd‐1 and Fas, and elevated triglyceride and bile acid levels are observed.^180^, ^181^ This has important implications in bile acid‐induced HCC.^182^ The gut microbiota has the ability to metabolize nutrients that contain methylamine, namely choline, lecithin, and l‐carnitine. As a result of this process, trimethylamine (TMA) is produced, which then undergoes a further transformation into TMAO through the action of flavin monooxygenases (FMO) located in the liver.^183^ Bile acids by gut microbiota activate FXR‐induced FMO3 expression and increase the development of hyperglycemia, hyperlipidemia, and atherosclerosis.^184^, ^185^ In other experiments, it was discovered that bile acids hindered the ability of DU‐145 migratory prostate cancer cells to adhere, migrate and invade. Both CDCA and DCA were found to destabilize HIF‐1α across all cells while effectively impeding crucial phenotypes related to cancer progression. This novel observation implies that bile acids have significant physiological implications in targeting hypoxic tumor advancement.^186^ With the rapid expansion of gut flora research, future studies will elucidate how the flora interacts with intestinal lipid metabolism pathways, undoubtedly leading to additional therapeutic options.

## 4 THE INFLUENCE OF MICROBIOTA ON TUMOR STROMA CELLS AND ANGIOGENESIS

### 4.1 Microbiota and tumor stromal cells

Fibrosis in some solid tumors is an important factor affecting the therapeutic effect of tumors. One of the causes of fibrosis in solid tumors (e.g., liver and pancreas) is the presence of a large number of stromal cells in TME, with stellate cells being the main component of stromal cells. Stellate cells are retinol‐storing cells that are quiescent under normal conditions; in the pathological state, they are activated and release extracellular matrix (ECM) components, including numerous critical regulators of fibrosis.^187^, ^188^ Microbiota and their derived metabolites can directly or indirectly activate stellate cells. *Stenotrophomonas maltophilia* exacerbates liver fibrosis by activating the TLR4/NF‐κB/NLRP3 pathway and promoting HSC expression of α‐smooth muscle actin (α‐SMA) and collagen I.^33^ HCC development in mice is stimulated by a number of inflammatory and tumor‐promoting mechanisms, all of which are upregulated by dysbiosis of the intestinal microbiota and hence elevated DCA levels.^79^ LPS activates PC cells via TLR4, NLRP3 inflammatory vesicle‐driven, expresses IL‐1β, and stimulates the activation and secretory phenotype of quiescent pancreatic stellate cells (PSCs).^189^ Microbially recruited immune cells, such as M2 macrophages, secrete transforming growth factor‐β (TGF‐β), which in advanced PC can effectively induce PSCs to secrete collagen α‐SMA, collagen I, and IV to exacerbate fibrosis.^190^, ^191^, ^192^ The TGF‐β1‐activated enhancer‐binding protein δ/HIF‐1α/hepatoma‐derived growth factor axis contributes to the antiapoptosis of PSC, leading to synthesis and deposition of ECM proteins.^193^ These fibrosis‐associated ECM, such as type IV collagen, periostin, promote cancer cell proliferation, migration, and apoptosis resistance, confer resistance to starvation and hypoxia, and limit the delivery of chemotherapeutic drugs to cancer cells.^194^, ^195^, ^196^

### 4.2 Microbiota and angiogenesis

Dysbiosis of the intestinal microbiota enhances intestinal permeability and chronic hypo‐inflammation features of inflammation, accompanied by elevated levels of IL‐6, IL‐1β, tumor necrosis factor alpha (TNF‐α), and VEGF‐A, ultimately exacerbating pathological angiogenesis.^197^ *C. difficile* generates two primary exotoxins, namely Toxin A and Toxin B, which induce VEGF‐A expression in endothelial cells through the HIF‐α, p38‐MAPK, and MEK1/2 signaling pathways and increase angiogenesis in human intestinal microvascular endothelial cells (HIMEC).^198^ LPS stimulates NOD‐like receptors (NLR) and TLR to increase micro angiogenesis and induce motility and proliferation of HIMEC.^199^ LPS‐mediated ERK phosphorylation leads to forkhead box protein C2 (FOXC2)‐ERK protein linkage, ERK‐dependent phosphorylation of FOXC2 serine and threonine, and additional delta‐like 4 activation (the master regulator of sprouting angiogenesis expression) gene expression to promote angiogenesis.^200^ LPS stimulates intestinal fibroblasts to produce insulin‐like growth factor‐1, IL‐6, PDGF‐BB, monocyte chemoattractant protein‐1 (MCP‐1), and macrophage inflammatory protein 1α, all of which may promote mucosal angiogenesis in a complementary manner.^201^ In vitro, low concentrations of the microbial derivative butyrate act proangiogenically via the receptor G‐protein‐coupled receptor 43.^202^ HPV‐16 oncoprotein induced HIF‐1α, VEGF, and IL‐8 expression in vitro and significantly enhanced angiogenesis in lung cancer cells, which may be associated with the involvement of PI3K/AKT signaling pathway and c‐Jun.^203^ Additionally, angiogenesis requires endothelial cells to interact with various cell types.^204^ The mesenchymal cells (MSCs) are in close proximity to the endothelial cells and generate factors that promote angiogenesis.^205^ TNF‐α induced IL‐8 production by HIMEC and intestinal fibroblasts, as well as upregulated VEGF‐R2 expression on HIMEC to promote angiogenesis by initiating endothelial cells and stimulating MSCs to generate angiogenic factors.^206^ Taken together, microbiota disorders may lead to an imbalance in the levels of pro‐ and antiangiogenic factors; these genes are tightly regulated in normal tissues and facilitate swift yet aberrant tumor angiogenesis.^207^

However, some scholars believe that tumor vessels exhibit tortuosity, dilation, and uneven distribution. The adjacent endothelial cells demonstrate loose attachment to each other, while the pericytes that surround the vessels generally remain detached from endothelial cells. Consequently, tumor vascular leakage occurs with dysfunctional blood flow due to irregular regulation of vascular permeability by pericytes.^208^ In vitro studies showed that some fungi significantly suppress HPV‐16 E7 oncoprotein‐induced angiogenesis in lung cancer.^209^, ^210^ Tumor angiogenesis does not necessarily equate to the increased blood supply to the tumor, as discontinuous basement membrane of immature neovascularization permits extravasation of plasma and protein, further increasing intratumor interstitial pressure, prolonged vascular collapse, and inadequate nutrient delivery.^211^ Furthermore, some tumors are unable to sustain vascular survival, which illuminates the presence of well‐formed, aggressive peripheral and central areas of necrotic hypoxia in certain highly angiogenic tumors.^212^ Thus, despite high vascular density, the neoplastic endothelium generated in the majority of malignancies is often deformed and dysregulated, lacks blood transport function, and is less effective in transporting oxygen and nutrient and drug delivery.^213^, ^214^

## 5 MICROBIOTA AND IMMUNITY

**FIGURE 4:**
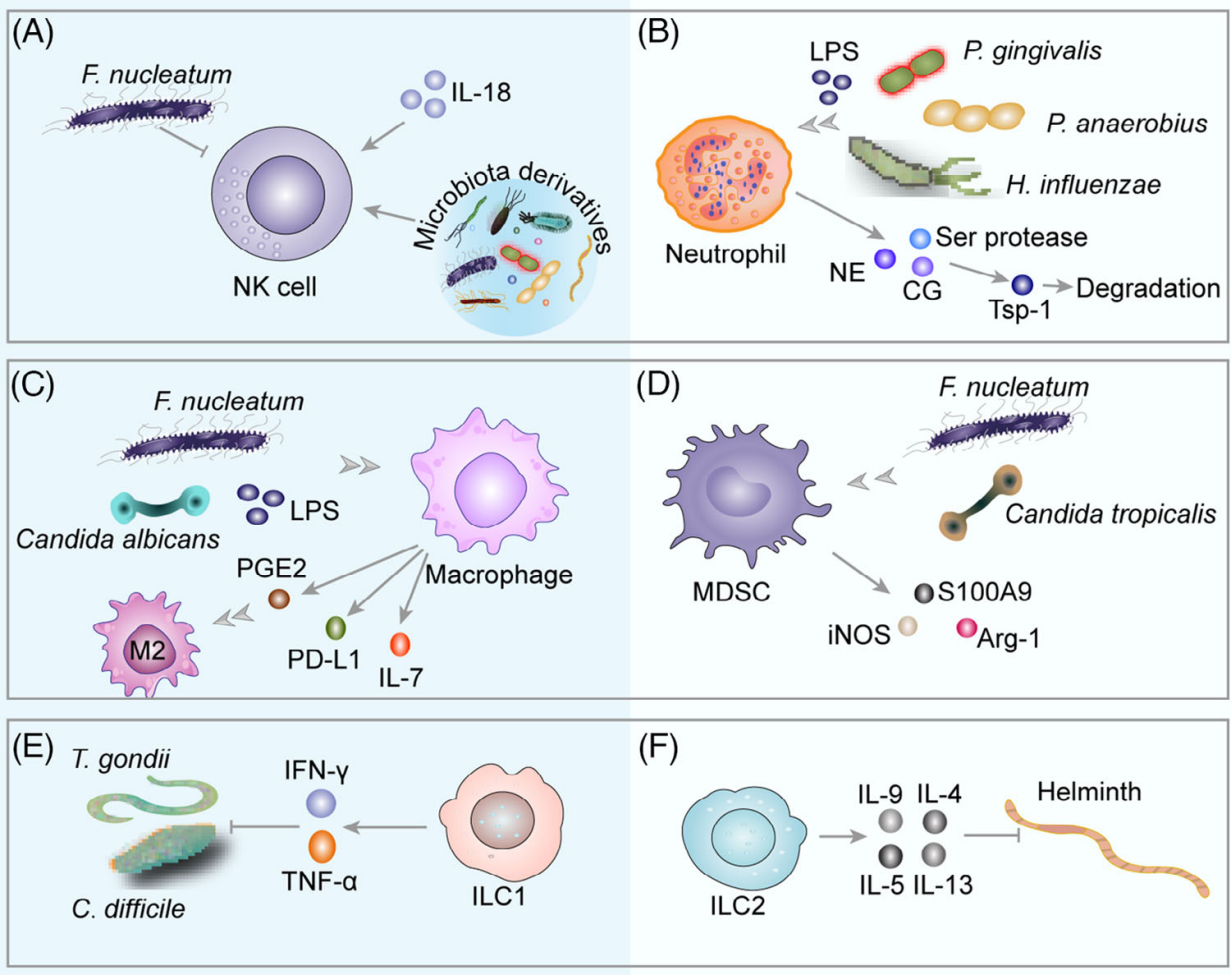
Innate immunity in microecology. (A) *F. nucleatum* promotes tumor cell metastasis by inhibiting NK cell recruitment. Conversely, other experiments have found that microbiota aid in recruiting NK cells and inhibiting tumor growth. Microbiota derivatives facilitate NK cell maturation and initiate their tumoricidal function by stimulating tumor cells to express IL‐8. (B) Microbiota and LPS stimulate TLR4, inducing NF‐κB activation and proinflammatory cytokine expression, recruiting neutrophils, releasing Ser protease, NE and CG, inducing the degradation of the antitumor factor Tsp‐1 protein, and enhancing the lung metastasis of tumor cells. (C) Microbiota and LPS recruit macrophages, secrete PD‐L1, and suppress immune effector T cells. *Candida albicans* regulates IL‐7 synthesis in macrophages via HIF‐1‐dependent glycolysis. PGE2 is produced by *Candida* or host cells and induces the polarization of M2 macrophages. (D) *F. nucleatum* and *Candida tropicalis* recruit and activate MDSCs, promote the expressions of S100A9, Arg‐1, and iNOS, and inhibit immune effector T cell function. (E) ILC1s produce IFN‐γ and TNF‐α and limit the proliferation of *T. gondii*, *C. difficile*. (F) ILC2s produce IL‐4, IL‐5, IL‐9, and IL‐13 against helminth. LPS, lipopolysaccharide; NE, neutrophil elastase; CG, cathepsin G; Tsp‐1, thrombospondin‐1; PD‐L1, programmed death ligand‐1; PGE2, prostaglandin E2; Arg‐1, arginase 1; IFN‐γ, interferon‐gamma; TNF‐α, tumor necrosis factor alpha.

**FIGURE 5:**
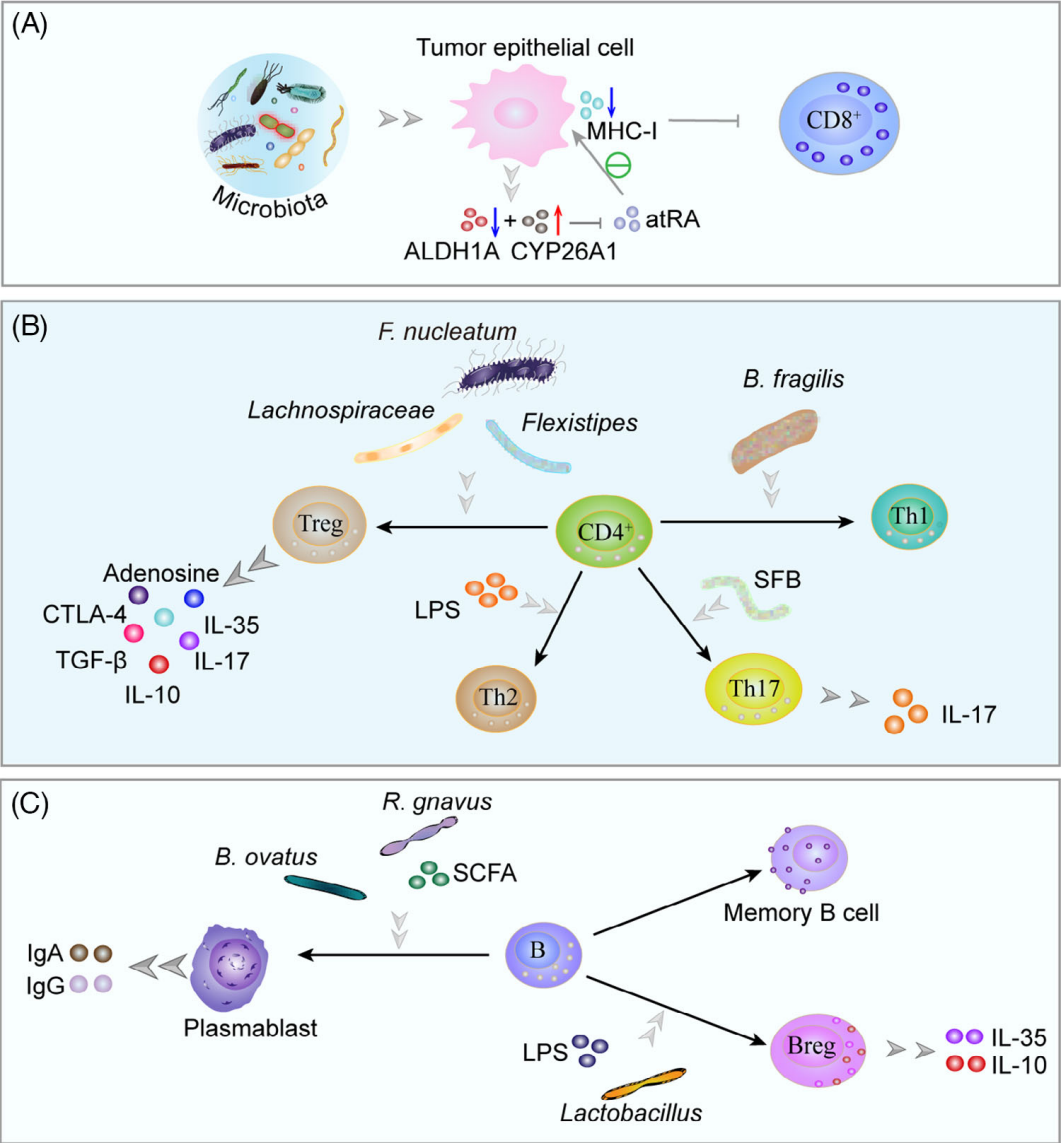
Adaptive immunity in microecology. (A) The microbiota reduces atRA by decreasing atRA‐synthesizing ALDH1A enzymes and increasing atRA‐catabolizing CYP26A1 enzymes, reduces MHC‐I expression in tumor cells and attenuates the sensitivity of CD8^+^ T cells to kill tumor cells. (B) *F. nucleatum*, *Lachnospiraceae*, and *Flexistipes* increase Treg cells, secreting immunosuppressive inhibitors such as adenosine, CTLA‐4, TGF‐β, IL‐10, IL‐17, and IL‐35. *B. fragilis* mediates the polarization of Th1. LPS promotes Th2 differentiation, while SFB induces the differentiation of IL‐17‐producing Th17 cells. (C) *B. ovatus, R. gnavus*, and SCFAs stimulate B cells, promoting plasmablast differentiation and the secretion of IgA and IgG. *Lactobacillus* and LPS promote Breg differentiation and the secretion of IL‐10 and IL‐35. atRA, all‐trans‐retinoic acid; ALDH1A, aldehyde dehydrogenase 1A; CYP26A1, cytochrome P450 26A1; MHC‐I, major histocompatibility complex class I; CTLA‐4, cytotoxic T‐lymphocyte‐associated protein 4; TGF‐β, transforming growth factor‐β; SFB, segmented filamentous bacteria; SCFAs, short‐chain fatty acids.

Microbiota, microbiota derivatives, and multiple chemokines recruit immune cell infiltration and activate members of the innate immune system (Figure 4), such as natural killer (NK) cells, neutrophils, macrophages, myeloid‐derived suppressor cells (MDSCs) and innate lymphoid cells (ILCs), and adaptive immune system (Figure 5), including T cells and B cells,^144^ which is a common and prominent feature of the microenvironment and should therefore be considered as a potential influence of the microenvironment on disease progression.^215^ Currently, how the microbiota affects the tumor immune microenvironment is in its infancy, with controversial results and still elusive mechanisms.

### 5.1 Microbiota and innate immunity in cancer

#### 5.1.1 NK cells

The gut microbiota and its derivatives are found to inhibit intratumor NK cell infiltration, thereby increasing tumor growth and metastasis.^216^, ^217^ *F. nucleatum* stimulates the hepatic immune response by recruiting MDSCs, reducing NK cell and T‐helper cell 17 (Th17) infiltration, and increasing hepatic Treg cell accumulation to promote CRC liver metastasis.^217^ Treg cells release TGF‐β, which drives the conversion of NK cells to ILC1 and assists tumor cells to evade the surveillance of innate immune system.^218^, ^219^ However, the microbiota‐mediated NK cell immunity mechanism remains contentious. Continuous exposure of the liver to multiple microbial products via portal circulation activates Fas ligand (FasL)‐sensitive CRC cells nucleotide‐binding domain, leucine‐rich‐repeat containing family, pyrin domain‐containing 3 (NLRP3) inflammatory vesicles and expresses IL‐18, which promotes NK cell maturation and initiates their tumoricidal function, and is an inhibitor of CRC metastatic growth in the liver.^220^ Gut microbiota mediates NK cell entry into the bloodstream via sphingosine‐1‐phosphate receptor 5 signaling, which is induced by CXCR3 to home to the bone marrow and inhibits the growth of bone metastatic malignant melanoma.^221^

#### 5.1.2 Neutrophils

Neutrophils are mostly found in the systemic circulation, are able to rapidly penetrate tissues, and are the first cells recruited to the site of an injury. Tumor‐associated neutrophils (TANs), the central granulocytes infiltrating the TME, promote tumor growth.^222^, ^223^, ^224^ LPS binding to TLR4 leads to NF‐κB activation and expression of proinflammatory cytokines, such as IL‐1β, TNF‐α, and IL‐6; generates an inflammatory microenvironment; recruits neutrophils; releases the Ser proteases, neutrophil elastase (NE), and cathepsin G; induces antitumorigenic factor thrombospondin‐1 protein degradation; and enhances tumor cell lung metastasis.^225^ When *P. gingivalis* was administered, tumor progression was increased in situ and subcutaneous PC mouse models that exhibited a neutrophil‐dominated proinflammatory TME. Mechanistically, intratumor *P. gingivalis* promotes PC development by enhancing the release of neutrophil chemokines and NE.^226^ *Peptostreptococcus* (*P*.) *anaerobius* activates the PI3K–AKT pathway in CRC, causing increased cell proliferation and activation of NF‐κB, increased IL‐10 and IFN‐γ expression, and recruitment of MDSCs, tumor‐associated macrophages (TAMs), and granulocytic TANs that are associated with chronic inflammation and tumor progression.^227^ Nontypeable *H. influenzae* stimulates LEC to express IL‐17C via TLR2/4, which in turn recruits TANs and promotes tumor growth.^228^ These protumor mechanisms may be owing to the existence of neutrophils, monocytes, and macrophages at the site of inflammation, which create oxidants and nitroso compounds that are known to be genotoxic and mutagenic.^86^ The protumorigenic effect of neutrophils in TME is controversial. In mice with inflammation‐induced and sporadic colon tumors, neutrophil depletion increased tumor growth, proliferation, and aggressiveness. Neutrophils slow the growth and progression of colon tumors by limiting bacterial populations and tumor‐associated inflammatory responses.^229^ The exact mechanism needs to be further elucidated.

#### 5.1.3 Macrophages

Microbiota signals recruit macrophages.^230^ TAMs are plastic and heterogeneous cell populations of TME and include two phenotypes, M1 and M2, that are responsible for up to 50% of certain solid tumors.^231^ TAMs stimulate tumor cell proliferation and survival, cultivate CSC, and support metastasis by maintaining the TME inflammatory environment creating an immunosuppressive microenvironment.^227^, ^232^ TAMs promote tumor cell proliferation and survival by maintaining the TME inflammatory environment creating an immunosuppressive microenvironment, nurturing CSC, and inducing progression and metastasis of several tumors such as gastric cancer, breast cancer, melanoma, bladder cancer, PC, and non‐small‐cell lung cancer.^233^, ^234^, ^235^ In early‐stage cancers, TAM has a proinflammatory M1 phenotype that stimulates increased antitumor activity by secreting proinflammatory factors IL‐1, IL‐6, and TNF‐α, which have antitumor actions.^236^ As the disease progresses, they exhibit a more M2 phenotype, secrete the anti‐inflammatory cytokine IL‐10, TGF, and promote tumor growth and invasion.^190^, ^237^ The main mediators of tumor‐induced polarization of macrophages from M1 to M2 types are chemokines and cytokines, such as prostaglandin E (PGE) 2, milk fat globule‐E8 (MFG‐E8), granulocyte/macrophage colony‐stimulating factor (GM‐CSF), CSF‐1, CCL5, IL‐4, IL‐6, and TGF‐β. Some noncoding RANs, such as lncRNA and miRNAs, can drive M2 macrophage polarization.^238^ Macrophages can boost the release of tumor‐promoting cytokines and immunosuppressive cytokines, including PGE2, VEGF, MCP‐1, IL‐6, IL‐1β, MMP‐9, TNF‐α, and cytidine deaminase (CDA), to enhance the pre‐metastatic fibrotic microenvironment, promote tumor cell proliferation, migration, invasion and EMT, and enhance tumor cell resistance,^239^, ^240^, ^241^, ^242^ and can express programmed death ligand‐1 (PD‐L1), bind to PD‐1 on T cells, deliver immunosuppressive signals and suppress immune effector T cells.^243^, ^244^ M2‐TAMs affect metabolite consumption and ROS production to directly suppress T cell activity.^241^ Furthermore, activated M2‐TAM releases various chemokines such as CCL17, CCL18, CCL20, CCL21, and chemokine receptor 4 (CCR4), CCR5, CCR6, and CCR10 to recruit and increase Treg cells and Th2 cells in TME, further damaging cytotoxic T lymphocytes (CTLs).^238^, ^245^ The polarization and function of macrophages are regulated by microbiota‐derived signals and the process is intricate.^246^, ^247^ *F. nucleatum* infection increases M2 polarization of macrophages in vitro and in vivo.^248^ Overgrowth of commensal fungal *Candida* in the intestine and plasma concentration of PGE₂, which induces polarization of pulmonary M2 macrophages.^249^ *Candida albicans* regulates IL‐7 synthesis in macrophages via HIF‐1‐dependent glycolysis, and IL‐7 stimulates IL‐22 synthesis in ILC3 via STAT3 and aryl hydrocarbon receptor (AhR), ultimately leading to elevated p‐STAT3 levels in epithelial cells and CRC development.^250^ Analysis of TAM phenotypes in a mouse model of PC revealed that microbial ablation caused a reduction in immunosuppressive CD206 M2‐like TAMs and an elevation in M1‐like TAMs expressing greater levels of major histocompatibility complex class II (MHC‐II), CD86, TNF‐α, IL‐12, and IL‐6.^38^ Interestingly, in the EL4 lymphoma tumor mouse model, the same population obtained from mice raised germ‐free showed a strong protumorigenic macrophage signature feature due to the lack of microbiota‐derived signaling, resulting in impaired intratumor IFN‐I signaling, mononuclear phagocytes are tilted toward suppressive macrophages. Tumor‐resistant macrophages require a microbiota‐derived stimulator of IFN genes (STING) agonist c‐di‐AMP.^251^ The gut microbial‐derived TMAO metabolite, which recruits more immunostimulatory macrophages and CD8^+^ T cells, slows tumor growth through an IFN‐I‐mediated manner.^252^

#### 5.1.4 MDSCs

MDSCs are a heterogeneous group of immature myeloid cells that promote immunosuppression and contribute to tumor development.^253^ Microbiota‐mediated TLR5 signaling drives tumor expression of IL‐6, which promotes MDSC mobilization and accelerates tumor growth.^254^ Bacteria of intestinal origin and LPS appear in the liver and stimulate CXCL1 expression in hepatocytes via a TLR4‐dependent mechanism, which in turn recruits CXCR2 MDSCs and promotes cholangiocarcinoma growth.^255^ Bacterial products regulate MDSC activity and promote CRC progression via NOD1.^256^ In recolonized germ‐free mice, colon tumor cells express CXCL1, CXCL2, and CXCL5 expression increase and recruit MDSCs, driving tumorigenesis.^257^ Dysregulated microbiota induces TLR4‐dependent amplification of hepatic MDSCs and suppresses T cell abundance.^258^ *F. nucleatum* enrichment in CRC tissue can selectively expand immunosuppressive myeloid cells, resulting in an immunosuppressive TME to further suppress T‐cell responses.^259^, ^260^ *Candida tropicalis* recruited and activated MDSCs in colon cancer tissue, promoted the expressions of S100A9, arginase (Arg)‐1, and iNOS, and inhibited CD8^+^ T cell or CD4^+^ T cell functions.^261^

#### 5.1.5 ILCs

ILCs are composed of three distinct groups: ILC1, ILC2, and the third ILC3, which play important roles in parasitic infections, inflammation, and cancer.^262^, ^263^ ILC1s produce IFN‐γ and TNF‐α and limit the proliferation of *Toxoplasma* (*T*.) *gondii*, *C. difficile* in the intestine.^264^, ^265^, ^266^, ^267^ ILC2s produce Th2‐cellassociated cytokines, such as IL‐4, IL‐5, IL‐9, and IL‐13, or epidermal growth factor receptor ligand amphiregulin and stimulate type 2 inflammation which is necessary for antihelminth immunity, allergic inflammation, and tissue healing.^268^, ^269^, ^270^ ILC3s can enhance antimicrobial immunity, chronic inflammation, and tissue healing by producing IL‐17A, IL‐17F, IL‐22, GM‐CSF, and TNF.^271^, ^272^ ILC3s mediate immune surveillance through DNA binding 2 (ID2)‐dependent IL‐22, sustaining appropriate colonization resistance against pathogens, such as *C. difficile*.^272^, ^273^

Furthermore, IL‐15 promotes the expression and cytotoxicity of ILC1 granzyme A, induces apoptosis in murine leukemic stem cells, maintains antitumor immunity, and correlates positively with patient survival.^274^, ^275^, ^276^ ILC2 triggered by IL33 expresses CCL5 selectively, attracts CD103+ DCs into tumor, and stimulates CD8^+^ T cells to induce therapeutic tumor immunity.^277^ However, additional tests showed that IL‐25 promoted intratumor ILC2 infiltration, retaining tumor‐infiltrating MDSCs to inhibit antitumor immunity and impair the survival of CRC patients.^278^ ILC3 is recruited and activated at the tumor site, where it promotes the generation of chemokine CCL20 and proinflammatory cytokine IL‐1β. ILC3 secretes chemokine CXCL10 inside the tumor, which encourages antitumor immune responses by recruiting CD4^+^ and CD8^+^ T cells.^279^ ILC3 deficiency increases metastasis of *Bacteroides*, *Erysipelotrichia*, and *Alistipes* in colon cancer, promotes colon cancer progression, and increases resistance to anti‐PD‐1 immunotherapy.^280^

### 5.2 Microbiota and adaptive immunity in cancer

#### 5.2.1 T cells

Microbiota stimulates CRC cells to produce multiple chemokines, recruiting CTLs, Treg cells, and Th cells.^281^ Intratumor microbiota mediates T cell infiltration, differentiation, and intricate immune effects. The microbiota has a crucial function in the maintenance of CD8^+^ T cell function.^282^ Intratumor microbiota such as *Lachnoclostridium genus*, *Gelidibacter*, *Flammeovirga*, and *Acinetobacter* induce tumor cells to secrete chemokines CXCL9, CXCL10, and CCL5, thereby recruiting CD8^+^ T cell infiltration.^283^ Mice with PC rich in CD8^+^ T cell infiltration and patients with cutaneous melanoma survived longer.^189^, ^283^ Unfortunately, the intratumor microbiota interferes more with CD8^+^ T cell infiltration. *F. nucleatum* reduces the accumulation of tumor‐infiltrating T cells and promotes tumor growth and metastatic development, the latter two of which are susceptible to antimicrobial treatment.^284^ Microbiota reduces all‐trans‐retinoic acid (atRA) by decreasing atRA‐synthesizing aldehyde dehydrogenase 1A enzymes and increasing atRA‐catabolizing cytochrome P450 26A1 enzymes, and promoting CRC progression. Microbiota elimination was followed by elevated atRA expression in tumors, which induced CD8^+^ T cell infiltration and MHC‐I expression by tumor epithelial cells, making CD8^+^ T cells more sensitive to tumor cell killing.^285^ After microbial elimination, a higher ratio of CD8^+^:CD4^+^ T cells was found in PC tissues, as indicated by Pushalkar et al.^38^ In addition, microbial elimination increased CD4^+^ T cell‐derived Th1 polarization and CD8^+^ T cell toxicity. After receiving antigenic stimulation, primary CD4^+^ T cells can separate into many subtypes of T cells under various conditions, including Th cells and Treg cells, which perform different functions.^286^ The proportion of Th1 and Th17 cells in the intestines of conventional and germ‐free mice fed stool from CRC patients was greater than that of mice fed stool from controls.^287^ Segmented filamentous bacteria and bacteria‐derived LPS stimulate colonic epithelial cells to secrete CCL2 and recruit monocyte‐like macrophage via TLR4 activation, and LPS further stimulates monocyte‐like macrophage to produce IL‐1β, which induces differentiation of IL‐17‐producing Th17 cells, IL‐17‐induced vascular growth and recruitment of neutrophils to produce a precancerous inflammatory environment to promote tumorigenesis.^288^, ^289^, ^290^, ^291^, ^292^ LPS‐induced DC amplification increased Th2 differentiation and promoted pancreatic carcinogenesis.^293^ Bacterial polysaccharide derived from *B. fragilis* is phagocytosed by DCs, presented to Th0 cells via MHC‐II, and finally, the IL‐12/STAT4 signaling pathway mediates the polarization of Th1.^294^ *B. fragilis* was found to enhance the effects of ipilimumab, a monoclonal antibody targeting cytotoxic T‐lymphocyte‐associated protein 4 (CTLA‐4), by boosting the Th1 immune response, thereby enhancing the efficacy of CTLA‐4 blockade immunotherapy.^295^ The increase of *Bacteroidetes* contributes to the appearance of Helios(‐) Treg cells, which decreases the lung response to allergens.^296^ *Fusobacterium* (*F*.) spp., *Bacteroides*, *Lactobacilli*, and *Flexistipes* increase in Treg cells.^297^ Foxp3+ Treg cells promote tumor progression through immune escape from PC.^298^ Elimination of *B. fragilis*, *Lachnospiraceae* reduced CD4+ CD25+ Foxp3+ Treg cell infiltration and inhibited CRC cell growth.^299^ Other experiments have found that depletion of CCR8+ Treg cells induces antitumor immunity.^300^

NKT cells are a specialized subset of T cells with T cell receptors and NK cell receptors that mediate antitumor immune surveillance.^301^ *Bacteroidetes*‐derived glycosphingolipids drive NKT expression of CCL5, which drives expansion and activation of hepatic leukocytes and regulates hepatic immunity.^302^ In contrast, microbiome‐derived butyrate increased hepatic NKT cells and decreased Treg cells, improving the anticancer immune response to CRC liver metastases.^303^ *C. scindens* boosted liver tumor growth by converting primary bile acids CDCA and TCA into secondary bile acids, lowering hepatocyte CXCL16 expression, and decreasing recruitment of CXCR6+ hepatic NKT cells.^304^ The intestine, skin, and lungs all have mucosal barrier tissues, and γδ T cells constitute a majority of resident T cells in these organs.^305^ γδ T have been hypothesized to have antitumor effects through unconventional specificity.^222^ γδ T cells percentage in CRC biopsies was significantly lower in all cases.^306^ However, *Herbaspirillum* and *Sphingomonadaceae* are examples of commensal microbiota that promote inflammation and tumor cell proliferation by stimulating Myd88‐dependent IL‐1β and IL‐23 production from myeloid cells, inducing proliferation and activation of Vγ6Vδ1 γδ T cell, producing proinflammatory cytokine IL‐17A through cell expression of transcription factor RORγt, and recruiting neutrophils.^307^

#### 5.2.2 B cells

There are five main stages in mammalian B cell differentiation: pro‐B, pre‐B, immature, naive, and mature B cells.^308^ In germinal centers, antigen‐activated B cells proliferate, express high‐affinity antibodies, and generate memory B cells. Although B cell proliferation is suppressed in hypoxic germinal center light zones, the metabolism of remaining B cells is reprogrammed to increase antibody affinity via iterative selection, allowing the most functional B cells to survive, mature, and proliferate.^309^ B cell developmental process is influenced by microbial colonization. Li et al.^310^ discovered that microbiota colonization modifies the B cell pool and its functional responsiveness, particularly at memory and plasma cell stages. *B. ovatus* and SCFAs stimulate B cells, promote plasma cell differentiation, and facilitated the production of IgG and IgA by decreasing AMP‐activated protein kinase (AMPK) activity, but increasing mTOR activity, while enhancing B cell glycolytic activity.^311^, ^312^ CRC biopsies revealed significantly increased immunity of B cells against commensal bacteria as well as *C. albicans*. Significantly higher effector memory B lymphocytes and plasmablasts were seen in CRC than in the healthy group.^306^ Symbiotic bacteria of the *Lachnospiraceae* family (e.g., *Ruminococcus* (*R*.) *gnavus*) stimulate plasma cells to secrete IgA and restrain tumorigenesis during CRC.^313^, ^314^ Bacteria in TME cause IL‐17 release, which stimulates the influx of intratumoral B cells, thereby promoting tumor growth and progression.^229^, ^315^ Microbial tryptophan catabolites, such as indole and its derivatives, can activate AhR, which then prevents B cells from differentiating in vivo into plasma mother cells and antibody‐secreting plasma cells.^316^, ^317^ AhR also contributes to regulatory B cells differentiation (Bregs), promotes IL‐10 secretion, and maintains an inflammatory environment.^318^ Additionally, IL‐10‐producing B cells are activated by intestinal bacteria through TLR2, MyD88, and PI3K pathways.^319^ *Lactobacillus* promotes B cell differentiation and expresses IL‐35 via 3‐indoleacetic acid in the presence of LPS.^320^ SCFAs are metabolites of gut microbiota that stimulate the differentiation of Bregs, the generation of IL‐10, and the production of IL‐1β and IL‐6 in the spleen and MLNs.^321^, ^322^ These factors create a protumor environment.^190^, ^323^, ^324^, ^325^, ^326^ The investigators also found that more effector memory B cells were present in the malignant basal‐like tumor tissue, a finding that may be attributable to excessive immune response and pathogen inflammation, hence promoting the proliferation of PC cells.^327^, ^328^

## 6 MICROBIOTA AND RESISTANCE TO CHEMORADIOTHERAPY

### 6.1 Microbiota and drug resistance

**FIGURE 6:**
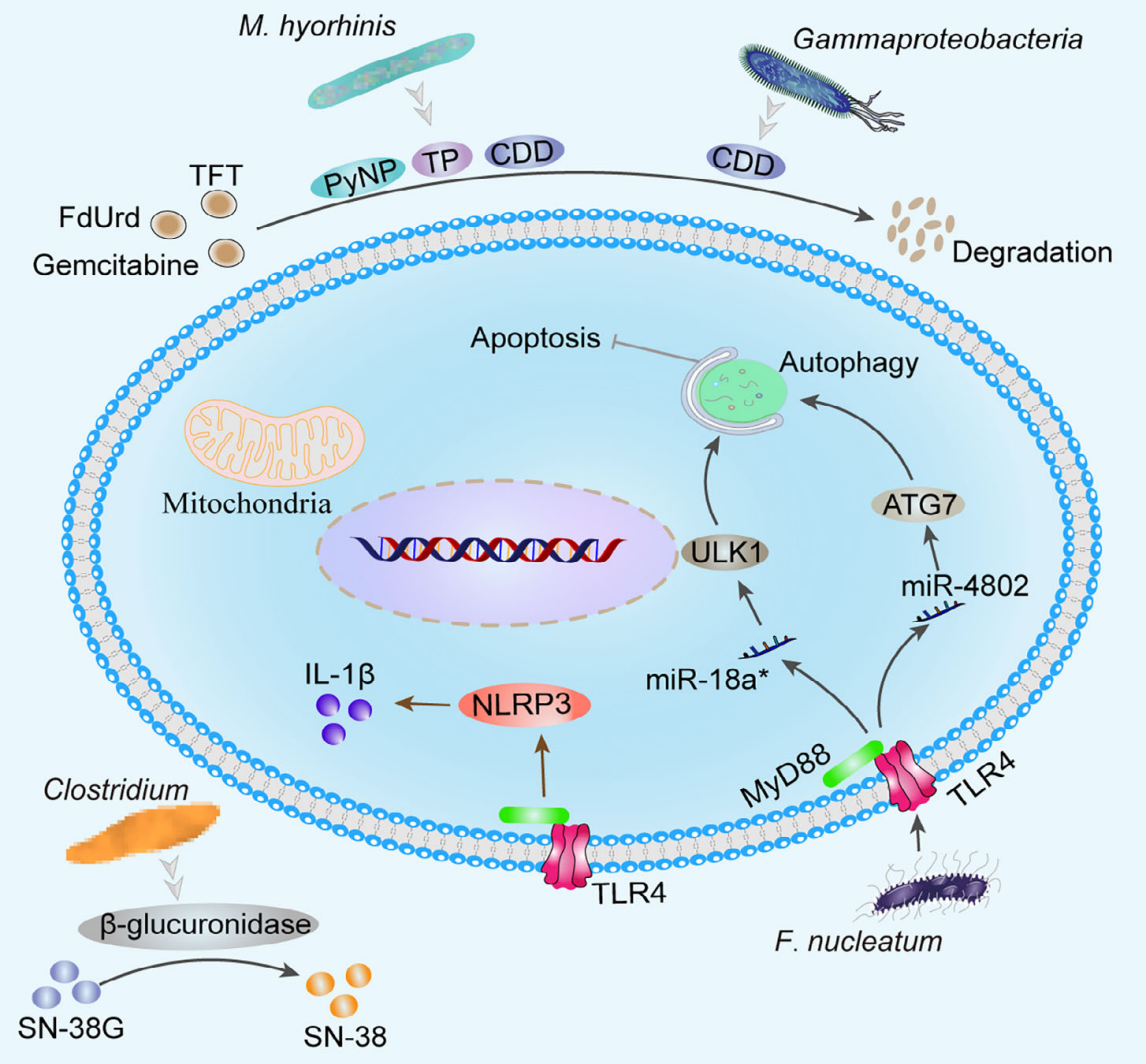
Effect of microbiota on cancer cell drug resistance. *F. nucleatum* targets TLR4 and MyD88 and specific miRNA (miR‐18a* and miR‐4802), respectively, targets ULK1 and ATG7, promoting autophagy activation in cancer cells, inhibiting apoptosis, and inducing resistance to oxaliplatin and 5‐FU. Microbiota dysbiosis induces the TLR4–NLRP3 inflammasome signaling pathway, promoting IL‐1β, which activates PSCs, ultimately exacerbating interstitial fibrosis and inducing chemoresistance. Additionally, enzymes produced by microbiota (e.g., CDD, PyNP, and TP) can directly convert drugs (e.g., FdUrd, FdUrd, and gemcitabine) into less toxic or nontoxic metabolites. β‐glucuronidase produced by *Clostridium* clusters XIVa and IV can convert SN‐38G back to cytotoxic SN‐38, causing severe diarrhea. ULK1, UNC‐51 like kinase 1; ATG7, autophagy‐related protein 7; NLRP3, nucleotide‐binding domain, leucine‐rich‐repeat containing family, pyrin domain‐containing 3; PSCs, pancreatic stellate cells; CDD, cytidine deaminase; PyNP, pyrimidine nucleoside phosphorylase; TP, thymidine phosphatase; SN‐38, 7‐ethyl‐10‐hydroxycamptothecin; SN‐38G, SN‐38 glucuronide.

The anticancer activity of chemotherapeutic drugs relies on the disruption of DNA integrity, that is, the enzymes used for DNA repair and synthesis. Generally, symbiotic microorganisms interact with chemotherapy medications by influencing drug metabolism (pharmacokinetics) and host immunity (i.e., pharmacodynamics). Microorganisms and their derivatives can transform medicines directly into nontoxic or toxicity‐reducing metabolites.^329^ *Mycoplasma* (*M*.) *hyorhinis* in cancer contains many nucleoside metabolizing enzymes such as thymidine phosphatase that degrade pyrimidine nucleoside analogs, including 5‐fluoro‐2′‐deoxyuridine (FdUrd), 5‐trifluorothymidine and 5‐halogenated 2′‐deoxyuridine to their inactive bases.^330^ *M. hyorhinis* produces CDD and pyrimidine nucleoside phosphorylase, which can determine gemcitabine to less cytostatic metabolite 2′, 2′‐difluoro‐2′‐deoxyuridine, which impairs anticancer effects of drugs.^331^ *Gammaproteobacteria* in TME can also produce CDD, leading to gemcitabine degradation and drug resistance.^332^ Besides, the microbiota antagonizes chemotherapeutic drug efficacy by activating cellular autophagy and remodeling the TME matrix. *F. nucleatum* targeted TLR4 and MyD88 innate immune signaling and specific miRNAs (miR‐18a* and miR‐4802), respectively, targeting UNC‐51 like kinase 1 and autophagy‐related protein 7 (ATG7) to promote autophagic activation in CRC cells to inhibit apoptosis and induce resistance to oxaliplatin and 5‐FU (Figure 6).^333^ Microbiota dysbiosis induces TLR4–NLRP3 inflammasome signaling pathway and promotes IL‐1β secretion by PC cells.^189^ IL‐1β induces chemo/immunotherapy resistance by activating PSCs, which can induce mesenchymal fibrosis.^334^

However, not all microbiota are detrimental to chemotherapy, and some can enhance the efficacy of chemotherapeutic agents. Scott et al.^335^ determined, using a simplified animal model of the nematode *Caenorhabditis elegans*, that *E. coli* vitamin B6 is needed for 5‐FU efficacy in *C. elegans*. Mechanically, PLP, the active form of B6, controlled glycine cleavage system, can be synthesized by *E. coli* and is responsible for mediating the efficacy of 5‐FU. Disruption of bacterial vitamin B6 production inhibits bacterial ribonucleotide metabolism and greatly antagonizes the efficacy of 5‐FU.^335^, ^336^ The SSL6 microbiota promotes sorafenib‐induced apoptosis of HCC cells Huh‐7 and MHCC97H by blocking CD47 and thereby downregulating PI3K/Akt‐mediated glycolysis.^154^ Alkylating chemotherapeutic cyclophosphamide (CTX) depletes immunosuppressive Treg and promotes Th1 cell development, hence inducing anticancer immunity.^337^, ^338^ *Enterococcus hirae* might elevate intratumoral CD8^+^/Treg cell ratio, while *Barnesiella intestinihominis* could boost the infiltration of IFN‐producing γδ T cells in cancer lesions and restore the CTX‐induced anticancer Th1 cell or CTL response, thereby controlling tumor progression.^339^ Microbiota also mediates host tolerance to chemotherapeutic agents. Irinotecan is a topoisomerase I inhibitor that is used as a first treatment for advanced CRC and is effective in a variety of cancers. Irinotecan is converted by carboxylesterase into 7‐ethyl‐10‐hydroxy camptothecin (SN‐38), an inhibitor of topoisomerase I that causes DNA replication and transcription arrest in tumor cells. Inactivation of SN‐38 to SN‐38 glucuronide (SN‐38G) occurs via glucuronidation catalyzed by various hepatic and extrahepatic diphosphoglucuronosyltransferase 1A isozymes.^340^, ^341^ The β‐glucuronidase produced by *C*. clusters XIVa and IV converts SN‐38G back to cytotoxic form SN‐38, resulting in severe diarrhea.^342^, ^343^ Therefore, the researchers propose to develop bacterial β‐glucuronidase inhibitors to improve the tolerance of irinotecan in cancer patients without killing the bacteria or harming mammalian cells.^344^ Such as *E. coli* βG‐specific inhibitor pyrazolo[4,3‐c]quinoline derivative (TCH‐3562), uronic isofagomine, and old drugs, including N‐desmethylclozapine, aspartame, and gemifloxacin, are anticipated to be a promising drug for prevention of CPT‐11‐induced diarrhea.^345^, ^346^, ^347^ Current research has focused on the relationships between intratumor microbiota and chemoresistance, although the mechanism underlying these interactions remain obscure. Chemotherapy also poses additional problems, as it contributes to the development of antibiotic‐resistant gut bacteria.^348^

### 6.2 Microbiota and radiation tolerance

**FIGURE 7:**
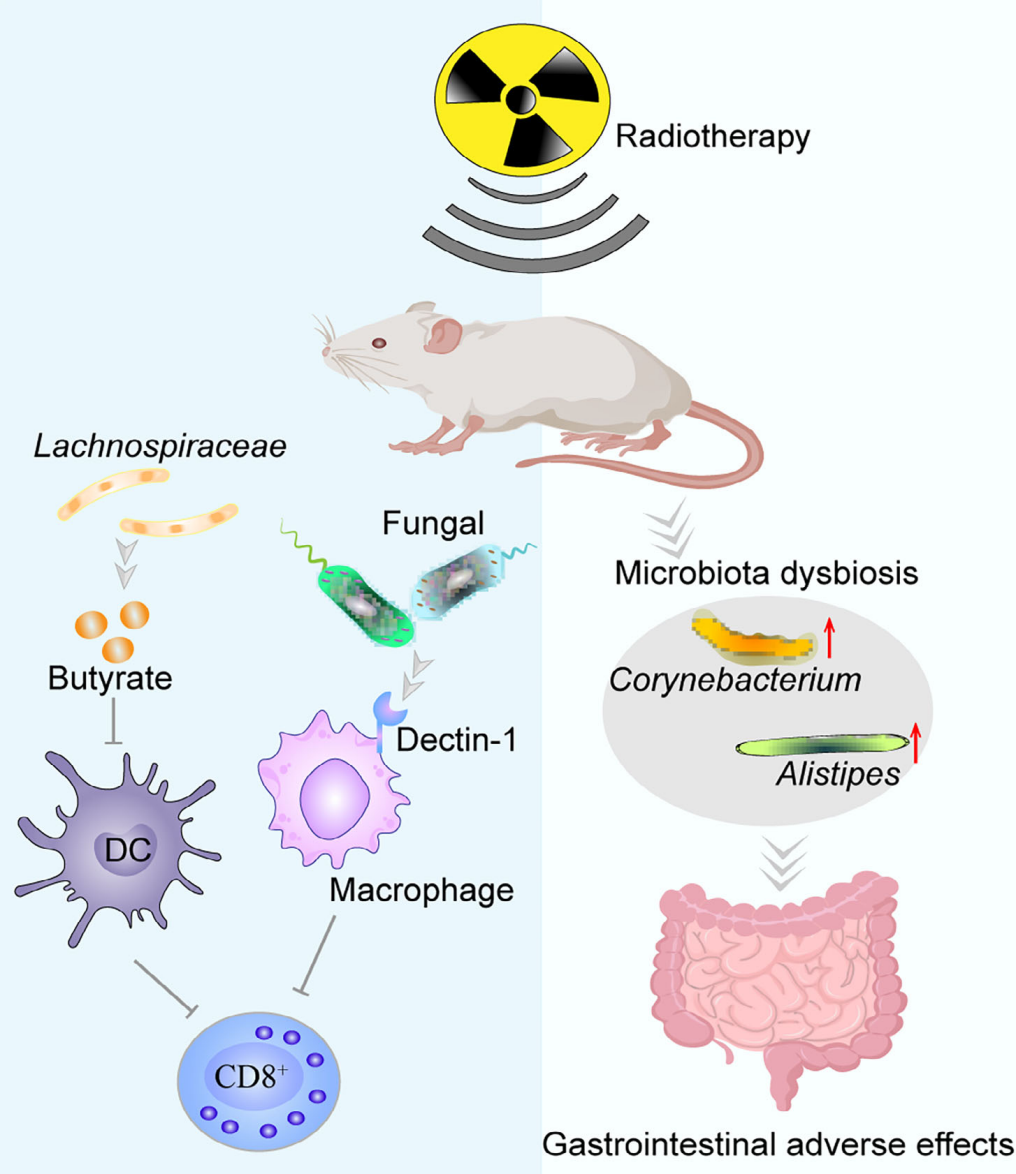
Effect of microbiota on tumor radiotherapy. SCFAs can impair the activity of APCs such as DCs, inhibit their antigen presentation function, limit CD8^+^ T cell infiltration and activation, and weaken the antitumor immune response induced by radiotherapy. Dysregulation of fungi can also reduce the antitumor immune response after tumor irradiation. Radiation treatment alters the structure of the host gut microbiota, increasing the levels of *Alistipes* and *Corynebacterium*, which can lead to gastrointestinal adverse effects. APCs antigen‐presenting cells, DCs dendritic cells.

Ionizing radiation's (IR) anticancer properties are partially mediated by the stimulation of innate and adaptive immunity.^349^, ^350^ The main tolerance of cancer cells to radiotherapy can be attributed to the disruption of intestinal microbiota. Gram‐positive bacteria and SCFA reduce the activity of antigen‐presenting cells (APCs) like DCs, inhibit their impaired antigen presentation function, limit CD8^+^ T cell infiltration and activation, and weaken radiotherapy‐induced antitumor immune response (Figure 7). Vancomycin enhanced RT‐mediated systemic anticancer effects by eliminating gram‐positive bacteria and reducing SCFA, enhancing the activity of DCs, increasing CD8^+^ T cell infiltration, and secreting IFN‐γ.^351^ Overgrowth of commensal fungi can reduce antitumor immunity after tumor irradiation. Macrophages expressing C‐type lectin receptor Dectin‐1 (sensing fungi) are recruited into tumor tissue to suppress adiation‐induced therapeutic antitumor immunity. Targeted commensal fungi enhance radiation‐induced antitumor immune responses by reducing macrophage‐mediated immunosuppression.^352^ Impaired anti‐HCC immunity is caused by dysbiosis of gut microbiota, which inhibits APC and suppresses effector T cell function via cGAS‐stimulator of STING–IFN‐I pathway.^353^ Oral administration of *Lachnospiraceae* produced butyrate suppressed STING‐activated IFN‐I expression in DCs by blocking phosphorylation of TANK‐binding kinase 1 and IRF3, thereby abrogating IR‐induced tumor‐specific cytotoxic T cell immunological responses.^354^ Meanwhile, radiation treatment alters the host gut microbiota and immune response after radiation therapy. For example, irradiation therapy significantly altered the bacterial composition of large and small intestines. Elevated levels of *Alistipes* and *Corynebacterium* in large and small intestines, respectively.^355^ Total body irradiation (TBI) impairs the gastrointestinal barrier in mice, allowing bacterial translocation associated with innate immune system activation, removing cytokine sinks and Treg cells. LPS via TLR4 signal improves the functionality of adoptively transferred CD8^+^ T cells.^356^ After TBI, neutrophils migrate from the damaged intestine to MLN, express MHC‐II, present host antigens, and participate in donor T cell expansion.^357^ Microbiota exacerbates tumor radiation tolerance partly by weakening the effective antitumor immunity. However, the specific molecular mechanisms by which radiotherapy interacts with the microbiota in tumors are unclear and require further study. In addition, high‐dose IR used during cancer radiotherapy is associated with the induction of hematopoietic, gastrointestinal, and cerebrovascular damage.^358^ Patient tolerance to radiotherapy can be improved by altering the host microbiota and its metabolites. Propionate and tryptophan metabolites derived from bacterial taxa *Lachnospiraceae* and *Enterococcaceae* attenuate hematopoietic and gastrointestinal syndromes and reduce proinflammatory responses.^359^ FMT elevated the level of microbial‐derived indole‐3‐propionic acid in the feces of irradiated mice, which activated pregnane X receptor/acyl‐CoA‐binding protein signaling and protected against gastrointestinal toxicity, demonstrating a lower system inflammatory level, recuperative hematopoietic organs, catabatic myelosuppression, improved gastrointestinal function, and epithelial integrity.^360^ Gut microbiota produced‐valeric acid supplementation increased survival, protected hematopoietic organs, and enhanced gastrointestinal function and intestinal epithelial integrity in mice that had been irradiated.^361^ Supplementation of microbiota‐derived PGF2α via the oral route activates the F‐prostanoid/MAPK/NF‐κB axis, promotes cell proliferation, and inhibits apoptosis, thereby reducing lung inflammation and improving pulmonary respiratory function after local chest irradiation.^362^

## 7 THERAPEUTIC APPLICATIONS

The microbiota in TME has an intricate relationship with tumorigenesis and development, and in particular, its interaction with innate and adaptive immune cells is particularly important. Ablation of pathogenic bacteria is associated with immunogenic reprogramming of the TME, including a reduction in MDSCs and an elevation in the differentiation of M1 macrophages, promoting Th1 differentiation and CD8^+^ T cell activation.^38^ Microbiota‐derived SCFA butyrate directly improves antitumor cytotoxic CD8^+^ T cell responses in vitro and in vivo in an ID2‐dependent manner by promoting cellular metabolism, enhancing the memory capacity of activated CD8^+^ T cells, and promoting the IL‐12 signaling pathway.^363^, ^364^ Thus, microbiota‐related therapies have great potential in cancer treatment (Table 2). Relevant microbiota‐based therapeutic measures are summarized as follows, including biocarrier, probiotic, prebiotic and synbiotic, and antibiotic applications, as well as dietary modification, defensins, oncolytic virotherapy, and fecal microbiota transplantation.

**TABLE 2:** Clinical trials of microbial‐related applications in various tumors.

Intervention/treatment	Microbial	Cancer type	Phase	Identifier	Therapeutic strategy
ADI‐PEG 20	Mycoplasma	Advanced HCC	III	NCT01287585	Intramuscular
Bacillus Calmette‐Guérin (BCG)	*Mycobacterium bovis*	Non muscle invasive bladder cancer Limited‐stage small cell lung cancer	I III	NCT01498172 NCT00006352	Intravesical Intradermal
Talimogene laherparepvec (T‐VEC)	Herpes simplex virus‐1	Pancreatic cancer Triple negative breast cancer and colorectal cancer with liver metastases Melanoma and sarcoma	I I II	NCT00402025 NCT03256344 NCT03555032	Intratumoral Intrahepatic Intratumoral
L‐asparaginase	*Escherichia coli*	Ovarian cancer Leukemia	II ‐	NCT01313078 NCT00506597	Intramuscular or intravenous
*Clostridium novyi‐NT* spores	Anaerobic bacteria	Solid tumor malignancies	I	NCT01924689	Intratumoral
*Salmonella* bacteria (VNP20009)	Facultative anaerobe	Cancer Neoplasm Neoplasm metastasis	I	NCT00004988	Intravenous
*Listeria monocytogenes*	Facultative anaerobe	Prostatic neoplasms	I	NCT02625857	Intravenous
*Clostridium butyricum*	Probiotics	Colorectal cancer	II	NCT00936572	Oral
*Clostridium butyricum*	Probiotics	Lung cancer	I	NCT02771470	Oral
Inulin and fructo‐oligosaccharide	Prebiotics	Endometrial neoplasms	–	NCT01549782	Oral
Date fruit‐Ajwa variety	Prebiotics	Colon cancer	–	NCT02288611	Oral
Oat bran and blueberry husks	Synbiotics	Rectal cancer	–	NCT03420443	Oral
LactoFos	Synbiotics	Head and neck cancer	–	NCT02654652	Oral
SMT04 formula	Synbiotics	Colorectal neoplasms	–	NCT05592886	Oral

*Data sources*: clinical registration website.

### 7.1 Biocarrier applications

Some bacteria have the ability to be drawn to the hypoxic core of cancer cells, where they can proliferate and spread. The use of microorganisms to target the hypoxic zone of cancer might be a viable cancer therapy technique. Spores of *C. novyi‐NT* significantly improved the efficacy of radiotherapy in various mouse models, such as biliary, colorectal, melanoma, and squamous cell carcinoma.^365^ Attenuated pathogenic facultative anaerobic *S. typhimurium* VNP20009 decorated by heptamethine cyanine dyes NHS‐N782 and extra terminal domain (BET) protein inhibitor, JQ‐1 derivatives, invades into tumor cells, targets mitochondria in tumor cells, and downregulates PD‐L1 expression in TME to produce an effective and durable T cell immune response.^366^ Attenuated *S. typhimurium* (S.T.ΔppGpp) was used to engineer a new strain of bioluminescent bacteria that enhanced PDT, promoted the conversion of anti‐inflammatory macrophages (M2) to proinflammatory macrophages (M1), stimulated tumor NK cells, CD4^+^ Th cells and CD8^+^ T cells, reduce immunosuppressive Treg cells in TME, and upregulate the expression of various effector cytokines.^367^ Nevertheless, colonized *S. typhimurium* may recruit large numbers of neutrophils, which favor tumor growth.^368^, ^369^ Silver nanoparticles modified with group sialic acid selectively recognize l‐selectin on neutrophil surfaces, deplete neutrophils, allow migration of *S. typhimurium* to surviving tumor regions, and enhance tumor suppression by inducing apoptosis leading directly to tumor death.^370^ *E. coli* membrane vesicle is an excellent catalase delivery that delivers catalase to decompose H_2_O_2_ into oxygen to alleviate hypoxia and radiotherapy resistance.^371^

### 7.2 Probiotic applications

Probiotics are defined as “live bacteria that impart a health benefit on the host when administered in suitable levels” and mainly consist of *Lactobacillus* and *Bifidobacterium* species,^372^ but also strains that reduce intestinal inflammation, induce immune regulation or enhance intestinal barrier function, such as *Akkermansia muciniphila, Faecalibacterium prausnitzii*, and *Roseburia* spp.^372^, ^373^, ^374^, ^375^ Available findings suggest that probiotics may regulate tumorigenesis and progression by influencing the regulation of intestinal bacteria and their metabolism, inhibition of carcinogens or carcinogenic agents, immune pathways, and apoptosis. The absence of probiotic *Parabacteroides distasonis* accelerates the development of CRC.^376^ Enrichment of probiotic microorganisms and depletion of pathogenic microorganisms inhibit proliferation, migration, invasion, and colony formation of tumor cells.^377^ Probiotics can improve intestinal microbiota structure, reduce carcinogen production, enhance antitumor immune response, and improve chemoradiotherapy for tumor patients.

Probiotic supplementation can improve intestinal microbiota structure, mostly by increasing other probiotics and decreasing harmful pathogenic microorganisms.^378^, ^379^ *Pediococcus pentosaceus* SL4 expresses and secretes a small protein P8, which can regulate the structure of intestinal microbiota by increasing *Akkermansiaceae* and *Lactobacillaceae* and decreasing the pathogenic bacterium *Turicibacter*, thereby inhibiting the proliferation of colon cancer cells and secreting antimicrobial peptides that inhibit pathogenic bacteria.^380^ *C. butyricum* modulates gut flora structure, changes intestinal microorganisms composition, decreases the *Firmicutes/Bacteroidetes* ratio, raises the relative abundance of probiotics, decreases colitis, and decreases the incidence and size of CRC.^381^ *Lactobacillus gasseri* and *Lactobacillus plantarum* significantly inhibit *H. pylori* activity and the ability to adhere to human gastric epithelial cells through metabolites, such as organic acids and proteases.^382^ *Lactobacillus ferment*, *Lactobacillus acidophilus*, *Lactobacillus johnsonii* MH‐68, and *Lactobacillus salivarius ssp*. have similar effects.^383^, ^384^ *L. coryniformis MXJ32* increases the abundance of *Lactobacillus*, *Bifidobacterium*, *Akkermansia*, and *Faecalibaculum*, reduced some harmful bacteria abundance, such as *Desulfovibrio* and *Helicobacter*, and reshaped the structure of antitumor intestinal microbiota.^385^

Probiotics are beneficial in reducing the production of carcinogens. Some *Lactobacillus* may remove carcinogenic compounds ochratoxin A, zearalenone, mycotoxins, heterocyclic aromatic amines, polycyclic aromatic hydrocarbons, and phthalic acid esters from the media environment through direct physical binding of cell wall peptidoglycan, avoiding the recontamination of toxic secondary metabolites resulting from the degradation of carcinogenic compounds.^386^, ^387^, ^388^, ^389^ Another reason is that *Lactobacillus* can inhibit the production of carcinogenic substances through metabolisms, such as by reducing ornithine decarboxylase, β‐glucuronidase, β‐glucosidase, nitroreductase, and azoreductase, reducing the risk of carcinogenesis.^387^, ^390^, ^391^, ^392^, ^393^ Probiotics such as *L. plantarum* CRD7, *Lactobacillus brevis*, *Lactobacillus paracasei*, *Lactobacillus casei*, *Bacillus subtilis*, and *Pichia anomala* improve aflatoxin M1 and aflatoxin B₁ metabolism and several hepatic enzyme gene expressions.^394^, ^395^, ^396^ *Streptococcus thermophilus* secretes β‐galactosidase that suppresses cell proliferation, reduces colony formation, stimulates cell cycle arrest and apoptosis in cultured CRC, and retards the growth of CRC xenografts.^397^ *Paenibacillus* 79R4 has the ability to decrease the negative effects of nitrate supplementation in ruminants by boosting nitrite reduction, enhancing fermentation efficiency, and improving ruminal nitrite/nitrate detoxification.^398^

Probiotics can influence tumorigenesis and progression by regulating tumor molecular mechanisms. *L. salivarius* blocks rat colorectal carcinogenesis in both in vivo and in vitro by suppressing AKT phosphorylation and expressions of downstream cyclinD1 and COX‐2.^399^ *Lactobacillus cocktail* and *C. butyricum* promote cancer cell apoptosis and suppress cancer cell proliferation and EMT by inhibiting Wnt/β‐catenin pathway, NF‐κB pathway, and Smad3 pathway.^381^, ^400^, ^401^ By downregulating the expression of antiapoptotic Bcl‐2 and proto‐oncogene K‐ras and upregulating proapoptotic Bax and oncogenic p53, *Lactobacillus rhamnosus GG* decreases tumor burden and tumor multiplication.^402^ *Bacillus polyfermenticus* inhibits the expression of ErbB2 and ErbB3 proteins and transcription factor E2F‐1, thereby inhibiting the expression of cyclin D1 and preventing colon cancer development.^403^ Exopolysaccharides from *L. plantarum* NCU116 inhibit cancer cell proliferation and induce apoptosis through TLR2 and c‐Jun dependent Fas/FasL‐mediated apoptotic pathway.^404^ Combination of ginger extract and *L. acidophilus* was effective in reducing COX‐2, iNOS, and c‐Myc expression.^405^

Probiotics also modulate innate and adaptive immunity in TME. *Lactobacillus bulgaricus*, *L. acidophilus*, and *L. plantarum‐12* reduced the levels of proinflammatory factors IL‐1β, IL‐6, IL‐8, IL‐17, IL‐23, and TNF‐α and increased anti‐inflammatory factor IL‐10 to alleviate intestinal inflammation,^378^, ^406^, ^407^, ^408^ which could be linked with the suppression of Smad7 and TLR4/NF‐κB signaling pathways.^379^, ^409^ Nevertheless, in another study, *L. acidophilus* could promote a Th1‐dominant immune response by stimulating proinflammatory cytokines expression, including IFN‐γ and suppress anti‐inflammatory cytokines expression, including IL‐4 and IL‐10 to enhance the antitumor immune response.^410^ *Bifidobacterium longum* or *L. gasseri* or both increase S‐phase DNA such as proliferating cell nuclear antigen (PCNA) and cyclin A synthesis, significantly promoted RAW264.7 macrophage proliferation, and increased phagocytic activity of peritoneal macrophages, thereby inhibiting 1,2‐dimethylhydrazine‐induced colonic tumorigenesis.^411^ *A. muciniphila* or Amuc_1100 supplemented could reduce CTL and CD16/32 macrophages infiltration in the spleen and MLN to alleviate colitis, but significantly reduced TAM recruitment and function and increased CTL tumor infiltration in colon cancer to blunt inflammation associated tumourigenesis.^412^ Short‐term supplementation with probiotics can increase polymorphonuclear phagocytic capacity or NK cell tumoricidal activity.^413^ Feeding beneficial microorganisms are sufficient to reduce the risk of cancer and obesity across generations.^414^ Intratumoral injection of *Bifidobacterium* can activate NK cells and inhibit tumor growth.^415^ *L. rhamnosus* strain GG inhibits the formation of neutrophil extracellular traps.^416^ *A. muciniphila* induces antigen‐specific T‐cell responses to regulate host immune function during homeostasis in vivo.^417^ *L. rhamnosus* aerosol reduced Treg cells in the lung, increased the joining chain of immunoglobulins and IgA, and reduced the number, diameter, and area of tumor nodules.^418^ Probiotics can induce activation of both CD4^+^ Th1 and CD8^+^ T cells in mice colon and human CRC, increase blood IFN‐γ levels, promote apoptosis in colon and breast cancer cells, and suppress cancer cell aggressiveness in vivo.^419^, ^420^ *L. casei* synergistically and selectively boosted IL‐12p70 expression by DCs and promoted polarization of naive CD4^+^ T cells toward a robust Th1 response without triggering Th17.^421^, ^422^ *Bifidobacterium* specifically enhances immunological checkpoint inhibitor (ICI) response and induces intratumor IFN‐γ‐CD8^+^ T cell accumulation.^423^ *A. muciniphila* restores the effectiveness of PD‐1 inhibition by boosting CCR9^+^CXCR3^+^CD4^+^ T lymphocyte recruitment to mouse tumor beds.^424^ In addition, the fine recognition mechanism of phage M13 filamentous phage selectively kills *F. nucleatum*, blocks the recruitment of immunosuppressive cells MDSC, induces DC maturation, and increases M1 phenotype TAM activation.^425^ Probiotics combined with chemotherapeutic agents like 5‐FU and gemcitabine can effectively boost antitumor effects through the use of polymers that are biocompatible and biodegradable with specific moieties or pH/enzyme sensitivity as carriers for targeted delivery.^426^, ^427^, ^428^

Probiotics can prevent or reduce radiotherapy‐induced nausea, vomiting, bloating, liver function impairment, and cardiac dysfunction and improve patient tolerance to radiotherapy without increasing clinically serious adverse events.^429^, ^430^, ^431^, ^432^, ^433^, ^434^ However, no evidence was found that probiotics prevent diarrhea or mucositis in advanced NSCLC patients.^435^

### 7.3 Prebiotic and synbiotic applications

Prebiotics are nondigestible substances that affect the content or activity of intestinal microbiota through the intestinal microorganisms metabolism, resulting in beneficial physiological effects.^436^ The main prebiotics that can be used as (candidate) prebiotics are fructo‐oligosaccharides, trans‐galacto‐oligosaccharides, soybean oligosaccharides, xylose oligosaccharides, arabinoxylan oligosaccharide, gentio‐oligosaccharides, lactulose, lactosucrose, inulin, and resistant starch,^437^, ^438^, ^439^ which are preferentially metabolized by probiotics into SCFAs, mainly acetate, propionate, and butyrate, and facilitate the impact on human health by improving resistance against pathogenic colonization, maintaining the integrity of mucus layer and colonic epithelium, lowering intestinal pH, modulating antitumor immunity and improving anticancer activity.^438^, ^440^ Among them, butyrate is the major SCFA in colonic bacteria metabolism and the preferred source of energy for colonic epithelial cells. 70–90% of butyrate is metabolized by colonic bacteria, while butyrate oxidation accounts for more than 70% of the oxygen required by human colonic tissues.^441^, ^442^ Butyrate is the preferred energy substrate, and it enhances the growth of normal colon gland cells while inhibiting the proliferation of colon adenocarcinoma, according to in vitro and in vivo investigations. The term “butyrate paradox” is used to describe this discrepancy.^442^ Butyrate stimulates p21 protein and mRNA levels, blocks the cellular G1 cycle, and inhibits cell proliferation.^443^ Butyrate significantly suppressed glucose transport and glycolysis in CRC cells by lowering the abundance of membrane GLUT1 and cytoplasmic glucose‐6‐phosphate dehydrogenase (G6PD) regulated by the GPR109a‐AKT signaling pathway.^444^ In the nucleus, butyrate acts as a histone deacetylase inhibitor to boost histone acetylation, reduce the expression of c‐Myc and PCNA (a nuclear protein involved in DNA replication and double‐stranded reconstruction) genes, induce apoptosis, and inhibit cell proliferation.^164^, ^392^, ^445^, ^446^ SCFA lowers intestinal pH, which can inhibit protease activity and thus affect protein fermentation. Limits the solubility of free bile acids and suppresses bacterial enzyme 7α‐dehydroxylase, that might decrease the synthesis of secondary bile acids and tumor‐promoting potential.^447^, ^448^ The activity and expression of nitroreductases, azoreductases, β‐glucosidases, and β‐glucuronidases were also inhibited in an acidic environment.^448^ A low pH environment inhibits the activity of enzymes responsible for ammonia release and reduces the production of ammonia, a postfermentation carcinogen for proteins and peptides.^449^ Furthermore, lactulose fermentation stimulates the growth of *Bifidobacteria* and increases the uptake of nitrogen by bacteria.^450^ SCFA greatly increased the generation of postswitch transcripts for IgG and IgA expression. Thus, SCFA stimulates B cells, promotes plasma cell differentiation, and facilitates the production of IgG and IgA by decreasing AMPK activity but increasing mTOR activity while enhancing B cell glycolytic activity.^311^ Butyrate also functions as a ligand for a specific GPR that has anti‐inflammatory properties, including the capability to trigger differentiation and expansion of Treg cells.^451^, ^452^, ^453^ By boosting IL‐12 signaling pathway, butyrate medication directly improves anticancer cytotoxic CD8^+^ T cell responses in vitro and in vivo in an ID2‐dependent manner.^364^ Pentanoate and butyrate increase the function of mTOR as a central cellular metabolic sensor and inhibit the activity of histone deacetylase class I in vitro treatment of CTL and chimeric antigen receptor T cells. This reprogramming raised effector molecules production such as CD25, IFN‐γ, and TNF and dramatically enhanced anticancer efficacy of antigen‐specific CTLs and ROR1‐targeting chimeric antigen receptor T cells in murine melanoma and PC models.^454^ However, SCFA can limit the anti‐CTLA‐4 activity. Butyrate suppressed anti‐CTLA‐4‐induced upregulation of CD80/CD86 on DCs and inducible T‐cell costimulator on T cells, as well as accumulation of tumor‐specific T cells and memory T cells, while boosting Treg cells in mice.^455^

Probiotics and prebiotics are ideally biocompatible and synergistic and are widely utilized as additives in food and pharmaceuticals, where they are combined to create microbiota‐regulating substances that are safe.^456^ Prebiotics selectively increase the activity and survival of probiotic microorganisms, which help maintain intestinal homeostasis, improve liver function, enhance immune regulation, prevent bacterial translocation, and reduce the incidence of nosocomial infections in surgical patients.^457^ Synbiotics intervention increased glutathione‐S‐transferase mu‐1 (GSTM1) and decreased MAPK9 gene expression in colon tumors, thereby attenuating the promotive and progressive effects of indole‐3 carbinol on colon carcinogenesis and reducing the risk of colon cancer in rats.^458^, ^459^ Prebiotics (dextran)‐encapsulated probiotic spores (*C. butyricum*) can effectively inhibit tumor growth in vivo by the mechanism that dextran is fermented by *C. butyricum* to produce SCFA, and this synbiotic can increase the overall richness of microbiota in the intestinal tract, increasing the number of SCFA‐producing bacteria.^460^ *Bifidobacterium breve* strain Yakult, *L. casei* strain Shirota, and galacto‐oligosaccharides reduced the frequencies of severe lymphopenia, febrile neutropenia and diarrhea in esophageal cancer patients receiving neoadjuvant chemotherapy (docetaxel, cisplatin, and 5‐FU chemotherapy regimens) by modifying the gut microbiota compared with the probiotic *Streptococcus faecalis* group alone.^461^ Our current comprehension of the mechanisms behind the beneficial effects of probiotics, prebiotics, and synbiotics is relatively superficial. Some probiotics and prebiotics cause host gastrointestinal discomfort, such as bloating, and wind up in pregnant women, newborns, and the elderly with potential probiotic infections.^462^, ^463^ To comprehend the interactions between microbiota, host, and prebiotic components, more clinical trials with bigger sample sizes are required.

### 7.4 Dietary modification

Our food impacts whether the microbiota creates metabolites that accelerate or retard tumor growth.^415^ Based on the host health benefits of probiotics, prebiotics, and synbiotics, natural products, and synthetic preparations can be administered orally to modulate microbiota structure, improve intestinal epithelial cell function and modulate the immune response.^464^ Dairy products containing probiotic strains, including fermented milk, buttermilk, milk powder, and yogurt, and nondairy products, including soy products, nutrition bars, cereals, and various juices, as appropriate means of delivering probiotics to consumers.^465^, ^466^ The diet rich in fiber can regulate the intestinal microflora after fermentation. Fermentation of *Phyllostachys edulis* shoots dietary fiber caused a reduction in intestinal pH, an elevation in *Alistipes* and *Lactobacillus*, and a significant decrease in *E. Shigella*, *Enterococcus*, as well as *Proteus*.^467^ *C. cluster* IV and XIVa are colonic bacteria that ferment dietary fiber into SCFA, butyrate, which exert tumor‐suppressing characteristics via several mechanisms.^164^, ^468^ Butyrate also aids in the maintenance of epithelial barrier function, which is crucial for preventing inflammation mechanistically. Butyrate may aid in the restoration of tight junction barrier in inflammatory bowel disease by modulating the expression of claudin‐2, occludin, cingulin, and zonula occludens proteins (ZO‐1, ZO‐2).^469^ Another study showed that butyrate oxidation as an energy source stimulates a HIF‐1α‐based pathway to sustain barrier function. Bacterial‐derived butyrate affects epithelial O_2_ consumption and leads to the stabilization of HIF, a transcription factor that coordinates barrier protection.^117^ MC38 tumors from mice fed a high‐fiber diet contained more DCs and monocytes and fewer macrophages, leading to an enhanced spontaneous anticancer response. In addition, cdAMP, a STING agonist derived from the gut microbe *A. muciniphila* in mice fed a high fiber diet, enhanced IFN‐I production by intratumor monocytes, modulating the recruitment and activation of NK cells and improving antitumor response.^251^ Oral propolis stimulates the increased cytotoxic activity of NK cells and prevents cancer progression as an adjuvant therapy to chemotherapy.^470^ Left ventricular mass, arterial thickness and stiffness, the risk of stroke, and the severity of heart failure, are all increased by a high‐salt diet (HSD).^471^ However, in a recent study, HSD increased NK cell‐mediated tumor immunity by inhibiting PD‐1 expression and increasing IFN‐γ and serum hippate. Although HSD‐induced tumor immunity was diminished due to a reduction in the intestinal flora, FMT from HSD mice restored tumor immunity linked with NK cell function. HSD increased *Bifidobacteria* abundance, which improved intestinal permeability, leading to intratumor localization of *Bifidobacteria*, promoting NK cell function, and resulted in tumor regression.^415^ Conversely, a fiber‐free diet leads to degradation of the mucosal barrier and increases susceptibility to pathogen ingestion.^472^ Mice maintained on a high‐fat and high‐sugar had more severe liver inflammation, tumorigenesis, and neutrophil infiltration.^414^, ^473^ Compared with individuals who consumed many beans, vegetable soups, potatoes, cooked vegetables, and raw vegetables, those who consumed many French fries, snacks, dips, vegetable oils, red meat, processed meats, and potatoes had a significantly higher risk of developing PC.^474^ Dietary vitamin E is an effective antimutagenic agent, both against naturally occurring and NO‐induced mutations. Vitamin E may provide protection through two mechanisms: elimination of NO‐related genotoxic species and modification of neutrophil infiltration into tumors.^86^

### 7.5 Antibiotic applications

Antibiotics affect tumors by altering the microbiota, modulating the immune response, and their own drug metabolism. Quinolone eliminates the specific pathogenic bacterium *Klebsiella pneumoniae*, which facilitates the survival rate of PC patients.^475^ Amphotericin B ablation of *Malassezia* prevents PC growth in vivo.^37^ Ciprofloxacin increases cancer cell drug toxic response by eliminating CDD‐secreting pathogenic bacteria from the mouse colon TME.^332^ In the lungs of mice nebulized with vancomycin/neomycin, the reduction in bacterial load was associated with a reduction in Treg cells and an elevation in T and NK cell activation, which coincided with a significant decrease in lung metastases from melanoma B16. Treatment with bacterial isolates from antibiotic‐treated lungs also reduced lung tumor metastasis.^476^ Administration of the antifungal terbinafine reduced fungal load, fungal‐induced MDSC amplification, and tumor load. Mechanistically, terbinafine directly inhibits tumor cell proliferation by decreasing the ratio of nicotinamide adenine dinucleotide phosphate (NADP) to NADPH and inhibiting G6PD activity, thereby causing disruption of nucleotide synthesis, deoxyribonucleotide starvation, as well as cell cycle arrest.^477^ The chemotherapeutic drug such as gemcitabine is more effective when combined with antibiotics than when used alone.^478^ However, long‐term antibiotic therapy significantly decreased the number of NK cells, IFN‐γ production, and CD8^+^ T cells in wild‐type mice lungs, hence increasing lung cancer growth and progression.^479^ In mice treated with antibiotics, *Burkholderials* families (*Alcaliginaceae* and *Burkholderiaceae*) increased, while *Prevotellaceae*, *Rikenellacaea*, and *Helicobacteraceae* families decreased, reducing NK cells and promoting glioma growth.^480^ In addition, antibiotics can prevent chemotherapy‐induced diarrhea and improve patients' tolerance to chemotherapy. For example, the combination of cholestyramine/levofloxacin is effective in preventing delayed diarrhea in CRC treated with irinotecan.^481^ Antibiotic use can cause infections with drug‐resistant bacteria. Vancomycin‐mediated disruption of microbiota homeostasis and subsequent loss of colonization resistance can lead to vancomycin‐resistant *Enterococcus faecium* intestinal predominance, resulting in bloodstream infections in hospitalized patients.^482^ Long‐term antibiotic usage increases the risk of cancer and reduces ICI treatment efficacy. Penicillin, sulfonamides, cephalosporins, macrolides, quinolones, and tetracyclines increase the risk of esophageal, stomach, PC, lung, prostate, and breast cancer.^483^, ^484^ Antibiotic use may be related with unfavorable outcomes in cancer patients receiving ICI.^485^, ^486^, ^487^ In mice models of sarcoma, melanoma, and colon cancer, the combined use of ampicillin, colistin, and streptomycin and the use of imipenem alone reduced the anticancer effects of anti‐CTLA‐4 treatment.^295^ Vancomycin pretreatment worsened CTLA‐4 blockage‐induced colitis in mice, which may be related to a vancomycin‐induced reduction of *Bifidobacterium*.^488^ Similarly, antibiotics inhibited PD‐1 blockade's effectiveness in cancer patients.^424^, ^489^ This may be related to antibiotic‐induced dysbiosis of the intestinal microbiota.^490^, ^491^ Oral antibiotics cocktail alters the microbiota, decreases the abundance of probiotics, and alters microbial metabolism in the gut of oral squamous mice, increasing tyrosine and decreasing glutamate levels and promoting the development of oral squamous cell carcinoma.^492^ Supplementation with probiotics can restore antibiotic‐damaged gut microbiota to improve the efficacy and responsiveness of anti‐PD‐1‐based immunotherapies.^493^

### 7.6 Defensins

Defensins are mainly synthesized by Paneth cells, neutrophils and epithelial cells, and contribute to host defense. Their roles in cancer development and progression have been a topic of intensive research.^494^ The study showed that 82% of prostate cancers and 90% of renal cell carcinomas exhibit cancer‐specific human β‐defensin‐1 (hBD‐1) protein deficiency.^495^ Overexpression of hBD‐1 gene in renal cancer cells SW156 leads to caspase‐3‐mediated apoptosis.^496^ Similar results were found in basal cell carcinoma, oral squamous cell carcinoma and CRC.^497^, ^498^ These data suggest that hBD‐1 might act as a tumor suppressor. Some probiotics can induce the production of defensins in human host cells. For example, surface layer protein (SLP) of *Lactobacillus helveticus* SBT2171 stimulates hBD‐2 expression by activating c‐Jun N‐terminal kinase (JNK) signaling via TLR2 in Caco‐2 human colonic epithelial cells.^499^ *lactobacilli* and the VSL#3 bacterial mixture (three *bifidum* and one *streptococcus* species) upregulates hBD‐2 by inducing proinflammatory pathways including NF‐κB and activator protein‐1 (AP‐1) as well as MAPKs.^500^ The flagellin of probiotic *E.coli* Nissle 1917 promotes hBD‐2 expression through NF‐κB and AP‐1 pathways.^501^, ^502^ *B. subtilis* and selenium‐enriched *B. subtilis* increase intestine BD‐1 expression through the TLR2–NF‐κB1 signaling pathway.^503^ Defensins are essential for gut homeostasis and restoration of the gut microbiota.^504^, ^505^ However, other experiments have found increased defensins expression in lung cancer tissues.^506^ The hBD3 promotes the proliferation and invasion of oral squamous cell carcinoma cells by regulating the expression of NF‐κB p65 and its downstream c‐Myc and p21.^507^ Overexpression of hBD‐3 might have a tumor‐promoting effect or contribute to lymph node metastasis.^508^ A deeper understanding of the interactions between probiotics and defensins will facilitate a comprehensive analysis of the crosstalk between defensins and microbiota dysbiosis in various gastrointestinal diseases, which is critical for the treatment of gastrointestinal diseases.

### 7.7 Oncolytic virotherapy

Oncolytic virus (OV) is a novel immunotherapy that achieves antitumor effects through the dual mechanism of selective tumor cell killing and induction of systemic antitumor immunity.^509^ OVs can be divided into two main categories: natural OVs (e.g., reovirus and vesicular stomatitis virus) and genetically modified OVs (e.g., adenovirus, vaccinia virus and herpesvirus).^510^ To date, four OVs and one non‐OV have been approved globally for the treatment of cancer. In 2005, the world's first oncolytic adenovirus drug, recombinant human adenovirus type 5, was approved by the National Medical Products Administration of China, in combination with chemotherapy for the treatment of patients with advanced nasopharyngeal carcinoma. In recent years, recombinant human adenovirus type 5 has made remarkable progress in the field of solid tumors.^511^ Talimogene laherparepvec (T‐VEC) is a genetically engineered herpes simplex virus type 1 (HSV1) that preferentially replicates in tumor cells and induces an antitumor immune response. T‐VEC was approved by the United States Food and Drug Administration (US FDA) in 2015 for the treatment of advanced melanoma.^512^ After ECHO‐7 was approved, production was discontinued due to manufacturing issues. Teserpaturev, a third‐generation HSV1‐based OV, has been approved in Japan in 2021 for patients with malignant glioma.^513^ In 2022, the US FDA approved nadofaragene firadenovec, the first nontumorolytic adenovirus encoding IFNα−2b, for nonmuscle‐invasive bladder cancer that is unresponsive to bacillus Calmette‐Guérin.^514^ Compared with conventional chemoradiotherapy, OVs precisely lyse cancer cells by interacting with specific cellular receptors, by exploiting tumor suppressor gene defects, by downregulating antiviral pathways in tumor cells, or by designing viral vectors with specific gene knockouts, and have antitumor effects that include antiangiogenesis, reversed metabolic reprogramming, and catabolism of the ECM surrounding tumor.^510^, ^515^, ^516^, ^517^, ^518^

Furthermore, OV combined with small molecule inhibitors/modulators targeting oncogenic signaling pathways can effectively enhance antitumor immunity and therapeutic efficacy.^519^ However, due to the tumor heterogeneity, the OV combination therapy strategy faces serious challenges.

### 7.8 Fecal microbiota transplantation

FMT, in which a healthy donor fecal microbiota is implanted and used to restore normal gut microbial community structure and function and to resist pathogen colonization, is considered a promising and effective treatment for a variety of gastrointestinal or immune‐related disorders.^520^, ^521^, ^522^ For instance, *C. difficile*, a member of the normal human intestinal flora, can cause dysbiosis of intestinal flora when antibiotics are not used in a standardized manner. Drug‐resistant *C. difficile* can grow and multiply in large numbers, leading to diseases such as antibiotic‐associated diarrhea and pseudomembranous colitis.^523^ Competing with *C. difficile* for nutritional and colonization resources, interfering with its virulence factors, and directly killing *C. difficile* are all capabilities of healthy gut flora. Intestinal microbiota can impede *C. difficile* germination and nutritional growth by activating numerous host immune defenses and restraining *C. difficile* with secondary bile acids.^520^ FMT with typical feces affected the immunological environment and gut homeostasis, resulting in an increase in gut length as well as epithelial proliferation and migration. This was related to dramatic alterations in microbiota composition, such as an elevation in the relative abundance of *Desulfovibrio* and *Akkermansia*. However, conventional microbiota transplantation elevated MSI in the untransformed intestinal epithelium of MSH2‐Lynch mice, suggesting that microbiota composition affects the mutagenesis rate of MSH2‐deficient crypts with increased oncogenic potential.^524^, ^525^ Therapeutic FMT decreases colonic inflammation and initiates intestinal homeostasis restoration by activating multiple immune‐mediated mechanisms simultaneously, ultimately resulting IL‐10 generation by innate and adaptive immune cells, such as CD4^+^ T cells, iNKT cells, and APCs, and decreases the capability of DCs, monocytes, and macrophages to present MHC‐II‐dependent bacterial antigens to colonic T cells.^521^ Supplementation with a single bacterium, *A. muciniphila*, via fecal transplantation improves intestinal barrier function, reduces MDSC infiltration, and inhibits steatohepatitis activity.^258^ FMT from mice fed HSD restored tumor immunity related to NK cell function, elevated *Bifidobacterial* abundance, caused increased intestinal permeability, resulted in intratumor localization of *Bifidobacteria*, boosted NK cell function, and promoted tumor regression.^415^ Mice receiving short‐term survival FMT had increased CD4^+^ Foxp3+ and MDSC infiltration, whereas tumors from mice receiving long‐term survival FMT had considerably greater CD8^+^ T cells and activated T cells as well as higher serum IFN‐γ and IL‐2 levels in mice.^526^ FMT and anti‐PD‐1 modify gut microbiota and reprogram TME to overcome anti‐PD‐1 resistance in a subset of advanced PD‐1 melanomas.^527^ Irradiated male and female mice exhibited improved survival after FMT, increased peripheral leukocyte amounts, and enhanced gastrointestinal function and intestinal epithelial integrity. FMT maintained bacterial composition and mRNA and lncRNA expression profiles of the small intestine of host sex specifically. While boosting angiogenesis, sex‐matched FMT did not increase cancer cell proliferation in vivo; therefore, FMT may act as a therapeutic method to reduce radiation‐induced toxicity and enhance the prognosis of tumor patients following radiotherapy.^528^ Conventional FMT mainly transfers large intestine‐derived microbes and gene functions into the recipient intestine, whereas only a small number of small intestine‐derived microbes have been successfully transplanted. Compared with FMT, whole‐intestinal microbiota transplantation introduces more small intestine‐derived microorganisms (e.g., *Proteobacteria*, *Lactobacillaceae*, and *Cyanobacteria*) into the recipient intestine and associated microbial functions, which improves intestinal morphogenesis, reduces plasma concentrations of IL‐5 and TNF‐α and increases concentrations of anti‐inflammatory cytokine IL‐4 in mice, thereby reducing the systemic inflammatory response in recipients.^529^ The mechanism may be related to T‐cell proliferation, gene expression, and prevention of apoptosis.^530^ Nonetheless, the possible risks of FMT, including the possibility of pathogen transmission to the recipient, should not be ignored. In order to identify more effective medications and reduce treatment complications, patients should be stratified based on their particular microbiota.

## 8 CONCLUSIONS AND PERSPECTIVES

Despite substantial advances in understanding the importance of microbiota for human health, the potential impact of the microbiota on tumor growth, progression, and treatment response remained unclear until recently. Additionally, the fact that microbiota populations are large and symbiotic within the human body, influenced by factors like age, sex, immune competence, diet, climate, geography, and various other aspects, complicates the use of microbiota for precision therapy.^143^, ^531^ A thought‐provoking question is whether certain probiotics yield beneficial effects on the organism in specific environments, such as the gut, and whether their colonization in other tissues also offers benefits—this needs careful assessment.

The microbiota‐based development of biovectors, probiotics, prebiotics, and synbiotics, along with the use of antibiotics, dietary modifications, defensins, oncolytic virotherapy, and FMT, has proven effective in enhancing antitumor efficacy. Furthermore, there exist other intricate factors in the TME. During the transition from in vitro and animal models to the final patient treatment, we tend to overlook certain determinants of the effectiveness of a singular treatment, such as the hypoxic microenvironment in tumors. Owing to the intricate nature of tumor relationships, the adoption of combined multidisciplinary approaches to cancer treatment (particularly for refractory cancers), encompassing fields like biology, chemistry, materials science, mechanics, electronics, and artificial intelligence, becomes imperative.

It is crucial to establish ideal and pathologically compatible models. For instance, by regulating physiologically relevant oxygen gradients, the established microfluidic gut microarray allows for the extended co‐culture of living human intestinal epithelium with stable colonies of aerobic and anaerobic human gut bacteria.^532^ Organoid is a model capable of preserving genetic, proteomic, morphological, and pharmacological properties of parent tumors in vitro and allowing for unparalleled genomic and environmental manipulation.^533^, ^534^ Establishing a complex model of organoids containing cancer cells and stromal cells can simulate apparent tumor hypoxic microenvironment and is good for studying cell phenotype.^535^ Overall, compared with animal models, organoids are low‐cost, human‐derived, near‐physiological, and can be well suited for in vitro interventions, not only for drug toxicity prediction, new drug screening, and personalized therapy studies but also for host‐pathogenic organism interactions, which may provide a reliable model for microbiota and their derivatives to intervene in hypoxic TME.^536^ However, compared with traditional models, the fidelity, stability, reproducibility, scalability, and precise control of microenvironmental conditions have become problems that need to be overcome in the development of organoid technology. As innovative research continues to evolve, organ‐like models will gradually tend to improve or give rise to new models and new research tools. In the future, organoid models may be applied in combination with the advantages of traditional models to open up new avenues for human microbiota and tumor research.

## AUTHOR CONTRIBUTIONS

C. Z., G. D. F., and Z. W. C. conceived the review. C. Z., G. D. F., W. Z. F., L. X., D. S., H. J. J., and Z. W. C. undertook the initial research. C. Z. and G. D. F. were involved in writing; Z. W. C. reviewed the manuscript. G. D. F. contributed equally to this work and should be considered co‐first author. All authors have read and approved the article.

## CONFLICT OF INTEREST STATEMENT

The authors declare no conflict of interest.

## ETHICS STATEMENT

Not applicable.

## Data Availability

Not applicable.
